# CCL3 and IL‐7 Synergistically Enhance CAR‐T Efficacy in Solid Tumors

**DOI:** 10.1002/advs.75993

**Published:** 2026-06-22

**Authors:** Huanpeng Chen, Xiaoyun Luo, Huixin Gao, Yujing Ke, Yu Zhan, Xiaoting Xu, Xiaoxiao Xiong, Fengjiao Wei, Si Yang, Zhonghua Liu, Bolan Yu, Zhaofeng Huang, Yingjie Bian

**Affiliations:** ^1^ The First Affiliated Hospital of Guangzhou Medical University Guangzhou National Laboratory Guangzhou Medical University Guangzhou Guangdong China; ^2^ Guangzhou National Laboratory Guangzhou International Bio‐Island Guangzhou Guangdong China; ^3^ Department of Clinical Laboratory Guangzhou Women and Children Medical Center Guangzhou Medical University Guangzhou Guangdong China; ^4^ Institute of Human Virology Zhongshan School of Medicine Sun Yat‐Sen University Guangzhou Guangdong China; ^5^ Key Laboratory of Tropical Disease Control (Sun Yat‐sen University) Ministry of Education Guangzhou Guangdong China; ^6^ BioResource Research Center Key Laboratory for Major Obstetric Diseases of Guangdong Province The Third Affiliated Hospital of Guangzhou Medical University Guangzhou Guangdong China; ^7^ Laboratory Animal Center South China Agricultural University Guangzhou Guangdong China; ^8^ Medical school of Jiaying University Meizhou Guangdong China

**Keywords:** CAR‐T, CCL3, IL‐7, residential memory T cells, solid tumors

## Abstract

Chimeric antigen receptor (CAR) T cell therapy has demonstrated remarkable clinical efficacy, but challenges remain in the therapeutic application for solid tumors, primarily due to poor infiltration capacity, suboptimal activity, and tumor antigen escape. Here, we found that the chemokine CCL3 plays a significant role in modulating intratumoral T cell cytotoxicity. However, CCL3 alone is insufficient to sustain durable CAR‐T cell‐mediated tumor killing, largely due to tumor‐induced T cell death. In contrast, the combination of CCL3 with interleukin‐7 (IL‐7), a cytokine known to enhance T cell survival, exerted a potent synergistic effect. This combination significantly improved CAR‐T cell infiltration and longevity in solid tumors, leading to robust anti‐tumor efficacy without significant side effects or systemic toxicity. Mechanistically, CCL3 plus IL‐7 promoted RUNX3 expression and facilitated the differentiation of CD69^+^CD103^+^ tissue‐resident memory T (Trm) cells. Furthermore, treatment with CAR‐T cells co‐expressing CCL3 and IL‐7 (3P7‐CAR‐T) remodeled the immune landscape of solid tumors, marked by an increased infiltration of M1‐like macrophages and CD103^+^ migratory dendritic cells. These changes enhance antigen presentation, thereby promoting the priming of endogenous anti‐tumor T cell responses. Taken together, our results demonstrate that CCL3 synergizes with IL‐7 to augment CAR‐T cell memory and therapeutic effectiveness in solid tumors.

## Graphical abstract

Synergistic delivery of CCL3 and IL‐7 by CAR‐T cells overcomes the immunosuppressive tumor microenvironment by enhancing cell infiltration, survival, and local immune reprogramming. This also establishes positive feedback that amplifies anti‐tumor activity and memory responses in both CAR‐T and endogenous T cells, presenting a robust therapeutic strategy for solid tumors.

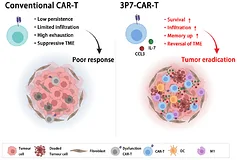

## 1 Introduction

Chimeric antigen receptor (CAR) T cell therapy has emerged as a transformative breakthrough in cancer immunotherapy, demonstrating remarkable clinical success for hematologic malignancies with sustained remission or clinical cure [1, 2]. However, the therapeutic potential of CAR‐T cells against solid tumors remains substantially limited, primarily attribute to the formidable barriers by the immunosuppressive tumor microenvironment (TME) [3, 4]. It critically impedes CAR‐T cell trafficking, persistence, and effector functions through multifaceted mechanisms including infiltration obstruction, metabolic competition, and functional exhaustion by inhibitory signaling [5]. To overcome these obstacles, strategic approaches must simultaneously address two critical aspects: 1) enhancing CAR‐T and intrinsic effector T cell homing capacity and persistence, and 2) developing innovative strategies to reprogram the TME. Consequently, the synergistic integration of these complementary strategies represents pivotal directions in translating CAR‐T therapy to solid tumors.

Chemokines orchestrate a wide range of T cell functions during immunity. In addition to the chemotactic properties, chemokines directly regulate T cell development, priming, and effector functions [6]. Studies have shown cooperation between CXCL9 and CCL5 in recruiting effector T cells into tumors [7]. Strategies have also been exploited to directly increase the level of CXCR3 ligands in preclinical models, which include plasmid‐borne CXCL9 [8], recombinant CXCL10 with adoptive cell therapy (ACT) [9], and intraperitoneal injection of oncolytic vaccinia virus expressing CXCL11 [10]. Besides, CCL19 and CCL21 have demonstrated enhanced CAR‐T efficacy through improved tumoral homing [11, 12]. However, given the diversity of the chemokine members [13], the optimal strategy for leveraging their functions to improve CAR‐T cell efficacy remains to be defined.

In this study, we identified CCL3 as a key gene associated with T cell cytotoxicity in solid tumors using transcriptomic profiling. Then, we engineered CCL3‐expression CAR‐T cells and demonstrated their capacity to enhance T cell tumor infiltration and cytotoxic function. However, CCL3 alone proved insufficient due to rapid T cell apoptosis induced by the hostile TME. Consequently, CCL3 plus interleukin‐7 (IL‐7), the central regulator of T cell survival and homeostasis, synergistically potentiate CAR‐T anti‐tumor activity and persistence. Mechanistically, we found CCL3 synergized with IL‐7 to promote RUNX3 expression and enhance CD69, CD103 levels for T cell memory and tumoral retention. The synergy didn't rely on tumor types, administration routes, target specificities, or intracellular signaling domains. Finally, CAR‐T cells co‐expressing CCL3 and IL‐7 (3P7‐CAR‐T) reprogrammed the TME, marked by an increased infiltration of M1‐like macrophages and CD103^+^ migratory dendritic cells. These changes enhanced antigen presentation, thereby promoting the priming of endogenous anti‐tumor T cell responses.

## 2 Results

### 2.1 Transcriptomic Profiling Identifies CCL3 as a Key Gene Associated with T Cell Cytotoxicity in Solid Tumors

**FIGURE 1:**
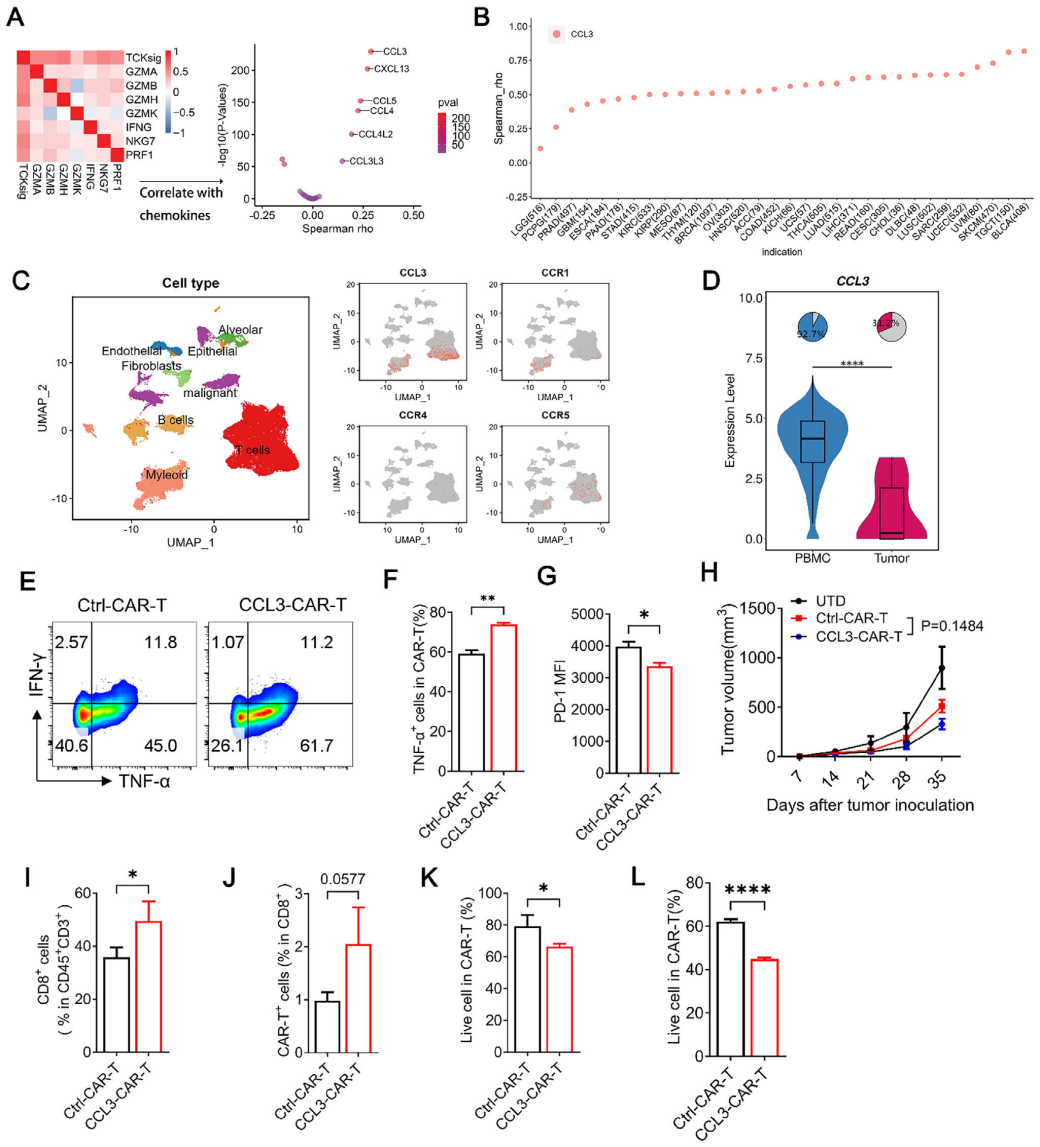
CCL3 Is Significantly Relevant to T Cell Killing and CAR‐T Anti‐tumor Efficacy. (A) Spearman correlation of chemokines and TCKsig expression in human lung cancer from the ArrayExpress database (E‐MTAB‐6149). (B) Spearman correlation of CCL3 with TCKsig expression across solid tumors from TCGA. (C) Levels of CCL3 and its receptors CCR1, CCR4, and CCR5 in distinct cell populations from human lung cancer database (E‐MTAB‐6149). (D) CCL3 levels in myeloid cells of PBMC and tumor tissue from Merkel Cell Carcinoma database (GSE118056). (E) Flow cytometry of IFN‐γ/TNF‐α expression in CAR‐T cells after two rounds of 96 h co‐culture with MC38‐Trop2 cells (N = 3). (F) The proportions of TNF‐α^+^ cells in CAR‐T cells. (G) The proportions of exhaustion marker PD‐1 in CAR‐T cells. (H–K) MC38‐Trop2 tumors in C57BL/6 mice (N = 4/group) were treated with Ctrl‐CAR‐T and 3P7‐CAR‐T, respectively. The tumor growth (H), proportions CD8^+^ T cells in CD45^+^CD3^+^ cells (I), proportions of CAR‐T cells in CD8^+^ cells (J), and cell viability in CAR‐T cells (K) were detected by flow cytometry. (L) The cell viability of CAR‐T cells after two rounds of 96 h co‐culture with MC38‐Trop2 tumor cells in vitro at a 1:2 effector‐to‐target (E: T) ratio (N = 3/group). Data are presented as mean ± SEM. Statistical significance: *p<0.05, **p<0.01, ***p<0.001, ****p<0.0001. Student's t test (D, F, G, and I–L), and two‐way ANOVA with Tukey's post‐test (H).

To investigate the association between chemokines and effector T cell functionality in solid tumors, we defined a T cell killing gene signature (TCKsig) composed of CD8A and effector molecules (GZMA, GZMB, GZMH, GZMK, PRF1, IFNG, and NKG7) (Figure 1A). TCKsig was then correlated with expression profiles of all 45 canonical chemokines using single‐cell RNA‐seq (scRNA‐seq) data from human lung cancer. Notably, CCL3 was identified as one of the most significantly associated chemokines and demonstrated a strong correlation with TCKsig (Figure 1A). To corroborate the findings, we further correlated chemokine profiles in human breast and liver cancers with TCKsig and observed a similar association (Figure S1A,B). Furthermore, pan‐cancer interrogation revealed significant CCL3‐TCKsig associations across all 32 TCGA solid tumor types (Figure 1B), including immunologically “cold” malignancies such as glioblastoma (ρ = 0.428, *p*<0.0001) and pancreatic adenocarcinoma (ρ = 0.467, *p*<0.0001). Thus, CCL3 is a key gene with T cell cytotoxicity in solid tumors.

Then, we further analyzed the correlation between CCL3 and survival outcomes across different cancers. The results revealed that CCL3 expression in tumors was positively correlated with patient survival (Figure S1C–F). Notably, CCL3 was significantly lower in tumor tissues compared to matched normal counterparts (Figure S1G–J). And, high CCL3 substantially reprogrammed the TME by promoting the infiltration of M1‐like macrophages and CD8^+^ T cells (Figure S1K–M). Receptor profiling via scRNA‐seq showed CCL3 receptors CCR1 and CCR5 were predominantly expressed on immune cells such as cytotoxic T lymphocytes (CTLs) (Figure 1C and Figure S1N), while CCL3 expression was mainly observed in myeloid cells including dendritic cells (DCs) and macrophages, and partially in CTLs (Figure 1C and Figure S1N). However, tumor‐infiltrating myeloid cells exhibited significantly reduced CCL3 secretion compared to their peripheral counterparts (Figure 1D). These findings suggested that endogenously promoting CCL3 expression in CD8^+^ T cells might represent a potential strategy to enhance CAR‐T cell efficacy.

Our previous studies have demonstrated the anti‐tumor function of CAR‐T cells targeting human Trop2 [14, 15]. To investigate whether CCL3 could enhance CAR‐T activity, we engineered CCL3‐CAR‐T cells. The T2‐m28z second‐generation CAR (Ctrl‐CAR) construct was linked to CCL3 using a self‐cleavable 2A peptide linker (Figure S1O). Retroviral transduction efficiencies for Ctrl‐CAR‐T and CCL3‐CAR‐T cells were both approximately 90% (Figure S1P). Results showed that CCL3 potentiated effector cytokines production (Figure 1E,F), while suppressing PD‐1 expression (Figure 1G) in CAR‐T cells. Adoptive transfer of CCL3‐CAR‐T cells partially enhanced in vivo anti‐tumor efficacy (Figure 1H), consistent with increased CD8^+^ T cells and CAR‐T cells infiltration (Figure 1I,J). However, CCL3‐CAR‐T cells exhibited compromised survival within tumors compared to Ctrl‐CAR‐T controls (Figure 1K). Besides, CCL3‐CAR‐T cells even showed attenuated anti‐apoptotic capacity when co‐cultured with tumor cells in vitro (Figure 1L).

Collectively, these data indicated that while CCL3 played a significant role in modulating intratumoral T cell cytotoxicity, it could not counteract tumor‐induced T cell death, thereby limiting the maintenance of durable CAR‐T cell activity.

### 2.2 CCL3 Plus IL‐7 Synergistically Potentiates CAR‐T Anti‐Tumor Activity

To simultaneously enhance CAR‐T cell tumor killing and persistence within the immunosuppressive TME, we propose a combinatorial strategy integrating CCL3 with IL‐7. IL‐7 has emerged as a central regulator of the survival and homeostasis of T cells, effector and memory T cell populations are highly dependent on the presence of IL‐7 for their persistence and survival [16]. We wonder whether CCL3 could synergize with IL‐7 to augment CAR‐T infiltration and persistence in the immunosuppressive TME. To address this, we generated a tandem construct encoding CAR, CCL3, plus IL‐7 (3P7‐CAR‐T), and corresponding controls, including parental CAR‐T (Ctrl‐CAR‐T), CCL3‐CAR‐T, and IL7‐CAR‐T (Figure S2A). The transduction efficiency of CAR‐T cells was comparable across all experimental groups (Figure S2B) and 3P7‐CAR‐T cells produced both IL‐7 and CCL3 when co‐cultured with Trop2‐expressing MC38 colorectal carcinoma (MC38‐Trop2) (Figure S2C,D).

**FIGURE 2:**
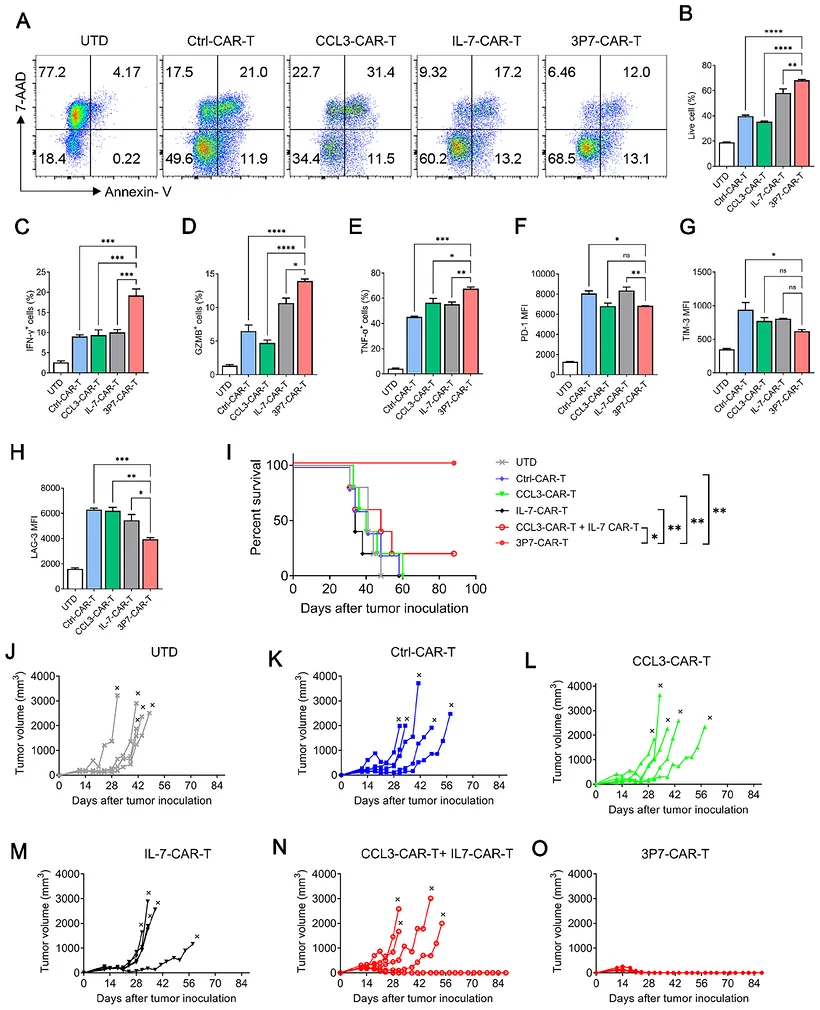
3P7‐CAR‐T effectively kills tumors both in vitro and in vivo. (A) Apoptosis of CAR‐T cells after two rounds of 96 h co‐culture with MC38‐Trop2 cells at a 1:2 effector‐to‐target (E: T) ratio. (B) Viability of CAR‐T cells (N = 3/group). (C–E) The expression of effector molecules, including IFN‐γ (C), granzyme B (D) and TNF‐α (E) in CAR‐T cells after two rounds of 96 h co‐culture with MC38‐Trop2 cells (N = 3/group). (F–H) The expression of exhaustion markers PD‐1 (F), LAG‐3 (G), and TIM‐3 (H) in CAR‐T cells after two rounds of 96 h co‐culture with MC38‐Trop2 cells (N = 3/group). (I) Survival curves of MC38‐Trop2 tumor‐bearing mice after CAR‐T treatment (N = 5/group). Cyclophosphamide was administered (*i.p*.) on day 11 post‐inoculation. Three days later, mice were *i.v*. administrated with 1 × 10^6^ Ctrl‐CAR‐T, CCL3‐CAR‐T, IL‐7‐CAR‐T, 3P7‐CAR‐T or a mixture of 0.5 × 10^6^ each of CCL3‐CAR‐T and IL‐7‐CAR‐T cells. Mice treated with untransduced T cells (UTD) after the CY administration were used as negative control. *P* values of Log‐rank (Mantel‐Cox) test were showed. (J‐O) The tumor volumes of each treated group are shown. “x”: dead or euthanized of the debilitated mouse. Statistical significance: *p<0.05, **p<0.01, ***p<0.001, ****p<0.0001. One‐way ANOVA with Tukey's post‐test (B–H) and Log‐rank (Mantel‐Cox) test (I).

We then investigated the effects of CCL3 and IL‐7 on CAR‐T cell viability, functionality, and exhaustion by in vitro experiments. The results demonstrated that while CCL3 or IL‐7 alone moderately enhanced CAR‐T cell effectors secretion or viability, their individual effects remained limited (Figure 2A–E). Strikingly, the combination of CCL3 and IL‐7 synergistically promoted CAR‐T cell survival (Figure 2A,B) and significantly upregulated the expression of key effector molecules, including IFN‐γ, TNF‐α, and Granzyme B (Figure 2C–E). Although Ctrl‐CAR‐T and 3P7‐CAR‐T cells exhibited equivalent short‐term cytotoxicity against MC38‐Trop2 cells (Figure S2E–H), 3P7‐CAR‐T significantly attenuated exhaustion during extended tumor co‐culture, which was evidenced by reduced expression of PD‐1, TIM‐3, and LAG‐3 (Figure 2F–H). These findings collectively indicated that CCL3 synergized with IL‐7 to enhance CAR‐T cell functionality and survival while mitigating exhaustion in vitro.

Subsequently, we conducted in vivo MC38‐Trop2 tumor‐bearing mice. On day 11 post‐tumor inoculation, mice were pretreated with cyclophosphamide (CY) and received injections of Ctrl‐CAR‐T, CCL3‐CAR‐T, IL7‐CAR‐T, or 3P7‐CAR‐T 3 days later. The 3P7‐CAR‐T group exhibited significant tumor suppression with prolonged survival, whereas CCL3‐CAR‐T and IL7‐CAR‐T therapies showed limited survival improvement (Figure 2I–O). In parallel, we evaluated the potential off‐target toxicities of 3P7‐CAR‐T cells. Analysis revealed no significant alterations in host body weight and absence of splenomegaly following 3P7‐CAR‐T cells transfer (Figure S3A–C). Systematic histopathological examination of major organs through hematoxylin and eosin (H&E) staining demonstrated preserved tissue architecture across all evaluated specimens, with no evidence of treatment‐related pathological lesions or aberrant lymphocytic infiltration (Figure S3D). These findings collectively indicated that the synergistic action of CCL3 and IL‐7 potentiated CAR‐T cell‐mediated tumor eradication without detectable toxicity.

### 2.3 CCL3 Plus IL‐7 Arise Synergistic Effect Intrinsically

**FIGURE 3:**
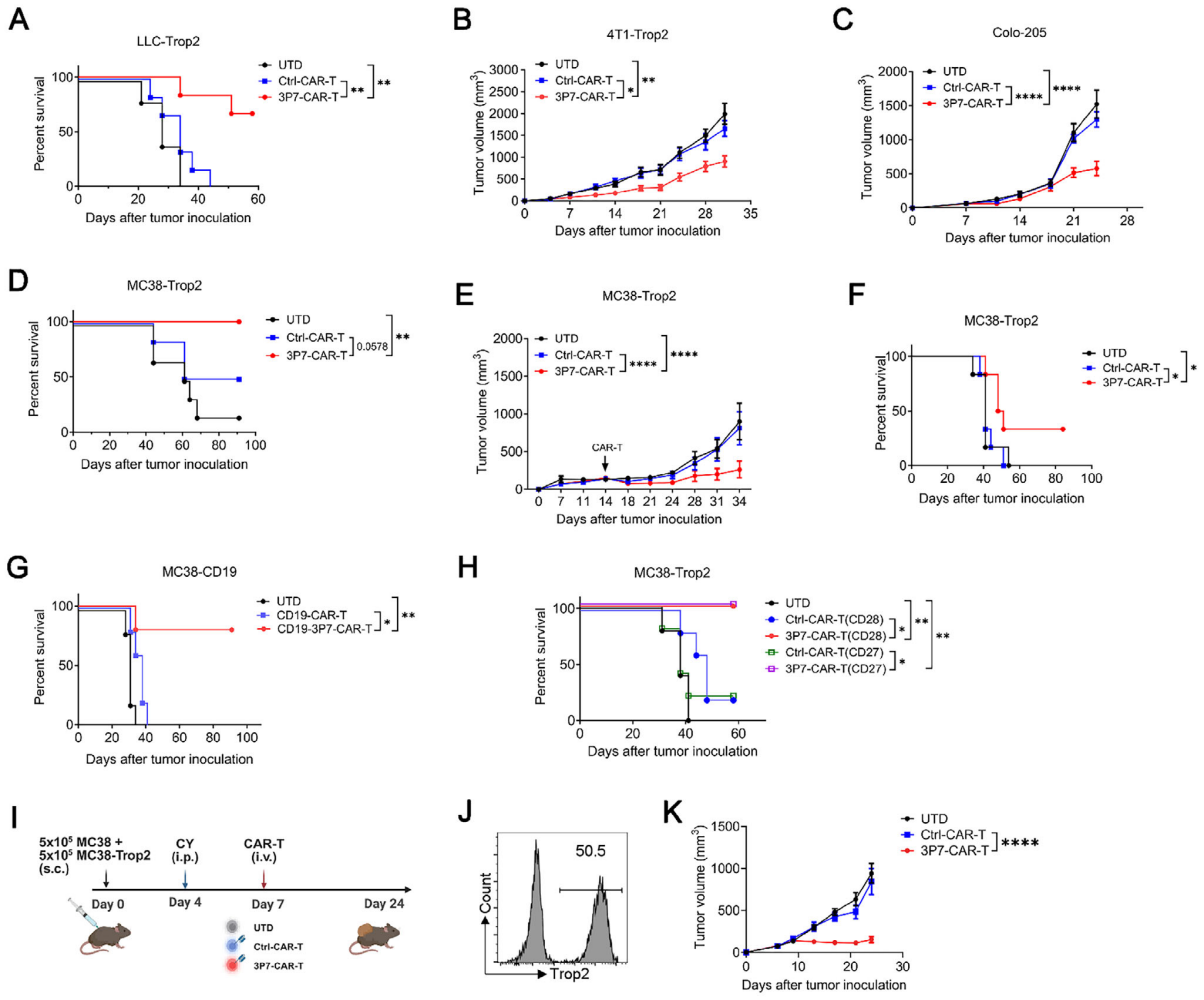
3P7 CAR‐T intrinsically promotes anti‐tumor responses. (A) Survival curve of LLC‐Trop2 lung cancer bearing mice after 3P7‐CAR‐T cells therapy (N = 5 for UTD, and N = 6 for Ctrl and 3P7‐CAR‐T group). (B) tumor growth of 4T1 tumors in BALB/c mice after 3P7‐CAR‐T cells therapy (N = 7/group). (C) tumor growth of human colon cancer in NCG mice after 3P7‐CAR‐T cells therapy (N = 6/group). (D) Survival curve of lung metastasis mice by intravenous injection of MC38‐Trop2 tumor cells, followed by 3P7‐CAR‐T cells treatment (N = 6/group). (E) Tumor growth of MC38‐Trop2 tumors followed by 3P7‐CAR‐T cells administration on day 14 without cyclophosphamide pre‐treatment (N = 6/group). (F) Survival curve of MC38‐Trop2 bearing mice followed by 3P7‐CAR‐T cells administration on day 14 without cyclophosphamide pre‐treatment. (G) Survival curve of MC38‐CD19 bearing mice, followed by CD19‐3P7‐CAR‐T cells treatment on day 14 (N = 5/group). (H) Survival curve of MC38‐Trop2 bearing mice receiving 3P7‐CAR‐T (CD27) cells (N = 5/group). (I) Experimental scheme of the mixed tumor model comprising MC38 and MC38‐Trop2 heterogeneous xenografts. (J) The Trop2 expression on the mixture tumor cells was assayed by FACS. (K) tumor growth of mixed tumors in C57BL/6 mice with the indicated CAR‐T cells treatment (N = 5/group). Survival rates were compared using Log‐rank (Mantel‐Cox) test. Data are presented as mean ± SEM. Statistical significance: *p<0.05, **p<0.01, ***p<0.001, ****p<0.0001. Two‐way ANOVA with Tukey's post‐test (B, C, E, and K) and Log‐rank (Mantel‐Cox) test (A, D, F, G, and H).

To elucidate whether the synergistic interaction of CCL3 plus IL‐7 operates specifically through an intrinsic mechanism rather than other factors, we conducted four complementary studies. First, we generated Trop2‐expressing Lewis lung carcinoma (LLC‐Trop2) and mammary carcinoma (4T1‐Trop2) models. Consistent with MC38‐Trop2 tumors, 3P7‐CAR‐T cells demonstrated complete tumor suppression and therapeutic efficacy against both lung and breast cancer subtypes (Figure 3A,B). Furthermore, in vivo experiments using Colo‐205 xenografts, a Trop2‐positive human colon carcinoma model, revealed that human 3P7‐CAR‐T cells significantly suppressed tumor progression (Figure 3C and Figure S4A–D), while conventional CAR‐T cells exhibited no therapeutic benefit. These data confirmed the tumor type‐independence of 3P7‐CAR‐T mediated synergy.

Second, we further evaluated the therapeutic efficacy of 3P7‐CAR‐T cells in a disseminated tumor model established via intravenous injection. It was observed that 3P7‐CAR‐T cells still improved the survival rate of tumor‐bearing mice (Figure 3D). Crucially, 3P7‐CAR‐T maintained efficacy regardless of cyclophosphamide (CY) pretreatment for lymphodepletion, as evidenced by comparable tumor suppression in CY pretreated vs. untreated hosts (Figure 3E,F). Thus, the synergy didn't depend on the administration and CY‐pre‐treatments.

Third, to assess antigen dependency, we generated CD19‐targeted 3P7‐CAR‐T cells. Administration of these CD19‐3P7‐CAR‐T cells significantly prolonged survival in MC38‐CD19^+^ tumor‐bearing mice, in stark contrast to the limited efficacy of conventional CD19‐CAR‐T cells (Figure 3G). This demonstrated that the synergistic therapeutic effect conferred by CCL3 and IL‐7 was consistently observed regardless of the CAR‐targeted antigen, indicating that the enhanced CAR‐T efficacy is not restricted to a specific antigen.

Last, we interrogated the contribution of intracellular domains to 3P7‐CAR‐T mediated synergy. We engineered 3P7‐CAR‐T cells by incorporating the CD27 co‐stimulatory domain—a signaling module with established T cell priming [17]. This structural exchange revealed that 3P7‐CAR‐T (CD27) demonstrated comparable anti‐tumor efficacy to their CD28‐containing counterparts 3P7‐CAR‐T (CD28) (Figure 3H). These findings conclusively demonstrate that the 3P7‐mediated synergistic mechanism is independent of intracellular signaling domains. Additionally, to determine whether the enhanced antitumor activity of 3P7 CAR‐T cells is restricted to tumors with homogeneous antigen expression, we established a mixed MC38 tumor model containing both Trop2‐positive and Trop2‐negative cells (Figure 3I,J). Our results demonstrated that, compared with the control group, 3P7‐CAR‐T cells exhibited similarly potent therapeutic efficacy in this heterogeneous tumor model (Figure 3K).

Thus, the 3P7‐CAR‐T mediated synergistic effects operated through an intrinsic pathway independent of tumor subtypes, CAR‐T target specificities, lymphodepletion status, and intracellular signaling domains.

### 2.4 CCL3 Synergized With IL‐7 to Promote T Cell Memory

**FIGURE 4:**
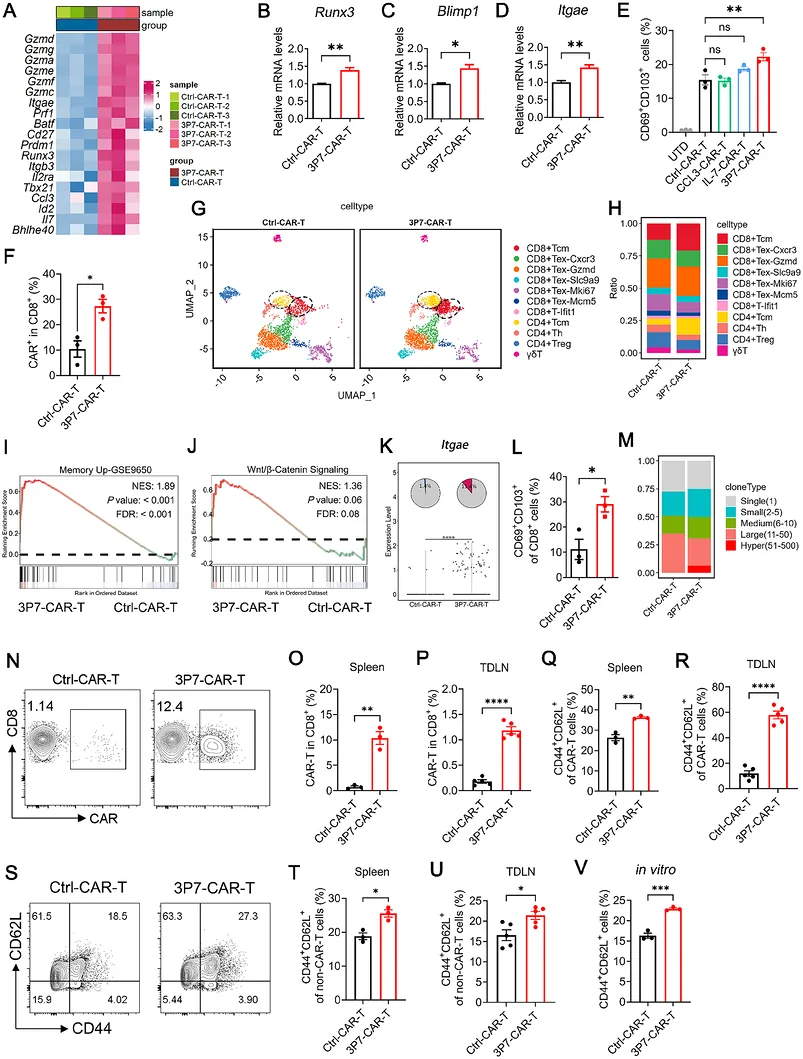
CCL3 synergizes with IL‐7 to drive memory T cell differentiation. (A) Heatmap of tissue‐resident memory T cell‐associated signature genes in 3P7‐CAR‐T vs. Ctrl‐CAR‐T cells after 48 h co‐culture with MC38‐Trop2 cells (N = 3/group). (B–D) mRNA levels of *Runx3* (B), *Blimp1* (C), *Itgae* (D) in CAR‐T cells were detected by real‐time PCR (N = 3/group). (E) The proportion of CD69^+^CD103^+^ CAR‐T cells after co‐culture with MC38‐Trop2 cells for 48 h (N = 3/group). (F) The proportion of CAR‐T cells in tumors was explored day14 after CAR‐T cell administration in MC38‐Trop2 tumor‐bearing mice (N = 3/group). (G) CD45^+^ cells were sorted from MC38‐Trop2 tumors treated with Ctrl‐CAR‐T and 3P7‐CAR‐T (N = 3/group), single cell RNA sequencing was performed. Uniform manifold approximation and projection (UMAP) analysis of non‐CAR‐T cells and 11 unique populations of T cells were identified. (H) The proportional changes by unique subcluster of T cells. (I and J) GSEA analysis of memory up (GSE9650‐Cluster 10&11) (I) and Wnt/β‐catenin signaling (J) in CD8^+^ T cells from 3P7‐CAR‐T cells compared with Ctrl‐CAR‐T cells. (K) The expression of *Itgae* on Tcm cells in tumors after CAR‐T cells treatment. (L) The proportion of CD69^+^CD103^+^ cells were detected by flow cytometry in MC38‐Trop2 tumor tissues (N = 3/group). (M) Clonotype size distribution of CD8^+^ T cells as determined by single cell TCR‐seq. (N‐P) Representative images (N), and the proportion of CAR‐T cells in the (O) splenocytes (N = 3/group), and (P) TDLN (N = 5) from MC38‐Trop2 bearing mice. (Q) The proportions of CD44^+^CD62L^+^ Tcm in CAR‐T cells of spleen (N = 3/group). (R) The proportions of CD44^+^CD62L^+^ Tcm in CAR‐T cells of TDLN (N = 5/group). (S) Representative images of CD44 CD62L staining in splenocytes from MC38‐Trop2 bearing mice. (T) The histogram shows the percentage of CD44^+^CD62L^+^ cells in non‐CAR‐T cells of spleen (N = 3/group). (U) The proportion of CD44^+^CD62L^+^ Tcm in non‐CAR‐T cells of TDLN (N = 5/group). (V) The proportions of CD44^+^CD62L^+^ Tcm in CAR‐T cells after co‐culture with MC38‐Trop2 tumor cells in vitro (N = 3/group). Data are presented as mean ± SEM. Statistical significance: *p<0.05, **p<0.01, ***p<0.001, ****p<0.0001. Student's t test (B–D, F, K, L, O–V) and one‐way ANOVA with Tukey's post‐test (E).

To investigate the underlying mechanism by which CCL3 synergizes with IL‐7 to enhance CAR‐T‐mediated tumor eradication, we performed RNA‐seq analysis for CAR‐T cells. Enrichment analysis revealed significant differences between 3P7‐CAR‐T and Ctrl‐CAR‐T cells in immune response‐related genes, particularly those governing cell migration, cell adhesion, and cell survival (Figure S5A). By comparing 3P7‐CAR‐T cells with control CAR‐T cells, we found significant upregulation of T cell residency and function‐related pathways in 3P7‐CAR‐T cells, along with elevated expression of transcription factors *Runx3*, *Blimp‐1*, and *Itgae* associated with residential memory T cells (Trm) differentiation (Figure 4A–D). These findings suggested that CCL3 synergized with IL‐7 to promote CAR‐T differentiation toward tissue‐resident memory T cells. We validated these findings through functional phenotyping. Flow cytometry confirmed enhanced differentiation of CD69^+^CD103^+^ tissue‐resident memory exclusively in 3P7‐CAR‐T cells (Figure 4E and Figure S5B). Consistent with this, we observed significantly increased intratumoral infiltration rates of 3P7‐CAR‐T compared to Ctrl‐CAR‐T cells (Figure 4F).

Given the paracrine secretion of CCL3 and IL‐7, we hypothesized that 3P7‐CAR‐T might similarly modulate endogenous tumor‐infiltrating lymphocytes. Thus, we performed scRNA‐seq with the tumor‐infiltration CD45^+^ lymphocytes. The results revealed significant expansion of endogenous T cells in 3P7‐CAR‐T treated tumors (Figure 4G,H). Subpopulation analysis demonstrated marked increases in both CD8^+^ and CD4^+^ memory T cells (Figure 4G,H and Figure S5C,D). Gene set enrichment analysis (GSEA) further revealed enhanced T cell memory in 3P7‐CAR‐T groups, as indicated by upregulation of memory and Wnt/β‐catenin signaling pathways (Figure 4I,J), but significant downregulation of T cell exhaustion markers (Figure S5E) [18]. Notably, the CD103 levels among intratumoral CD8^+^ T cells was significantly elevated in 3P7‐CAR‐T treated mice (Figure 4K). Flow cytometric validation also showed elevated CD69^+^CD103^+^ populations in tumor‐infiltrating non‐CAR‐T cells from 3P7‐CAR‐T‐treated mice (Figure 4L), paralleling the differentiation pattern observed in 3P7‐CAR‐T cells (Figure 4E). Moreover, single cell TCR sequencing indicated that 3P7‐CAR‐T treatment had markedly increased endogenous CD8^+^ T cells clonal expansion in tumors (Figure 4M).

We then assessed CAR‐T memory cells in the spleen and tumor‐draining lymph node (TDLN) after Ctrl‐CAR‐T or 3P7‐CAR‐T treatment. Flow cytometric analysis demonstrated significantly elevated frequencies of 3P7‐CAR‐T cells in both the spleen and TDLN (Figure 4N–P), which were predominantly of the Tcm phenotype (Figure 4Q,R). Notably, we also analyzed the endogenous (non‐CAR) T cells and found that they also exhibited significantly increased Tcm proportions (Figure 4S–U). However, no differences in memory phenotype were observed between 3P7‐CAR‐T and Ctrl‐CAR‐T cells prior to infusion (Figure S5F,G). In contrast, through in vitro co‐culture with tumors, we further demonstrated that CCL3 synergizes with IL‐7 to drive Tcm differentiation (Figure 4V). These findings collectively demonstrate that 3P7‐CAR‐T promotes memory T cell differentiation in both engineered CAR‐T and endogenous tumor‐infiltrating T lymphocytes.

### 2.5 3P7 CAR‐T Cells Promote Antigen‐Specific and Memory T Cell Responses to Control Tumor Recurrence

Antigen‐specific T cells are important for tumor control. To investigate whether 3P7‐CAR‐T therapy induces endogenous T cells with tumor antigen specificity, we performed three complementary analyses. First, single‐cell TCR repertoire analysis on tumor‐infiltrating lymphocytes isolated after 3P7‐CAR‐T treatment revealed significant clonal expansion. Among the top 10 TCRβ clonotypes that were highly enriched following treatment (Table S1), the hyperexpanded clone exhibited a TCRβ sequence that shares high amino acid identity with a previously reported p15E‐specific TCRβ in MC38 models [19]. Second, we isolated CD8^+^ T cells from TDLN of MC38‐Trop2 tumors treated with Ctrl‐CAR‐T or 3P7‐CAR‐T cells and co‐cultured with MC38 or MC38‐Trop2 cells. CD8^+^ T cells from the 3P7‐CAR‐T group, but not the Ctrl‐CAR‐T group, produced significantly higher IFN‐γ when co‐cultured with MC38 cells, indicating generation of endogenous tumor antigen‐specific CD8^+^ T cells (Figure S6A–C). Third, we directly assessed antigen‐specific T cells using p15E tetramer staining. The results showed that 3P7 CAR‐T therapy significantly increased the frequency of endogenous tumor antigen‐specific T cells in both TDLN and tumors (Figure S6D–G). Together, these results indicate that 3P7‐CAR‐T therapy induces the generation of endogenous tumor‐specific T cells.

**FIGURE 5:**
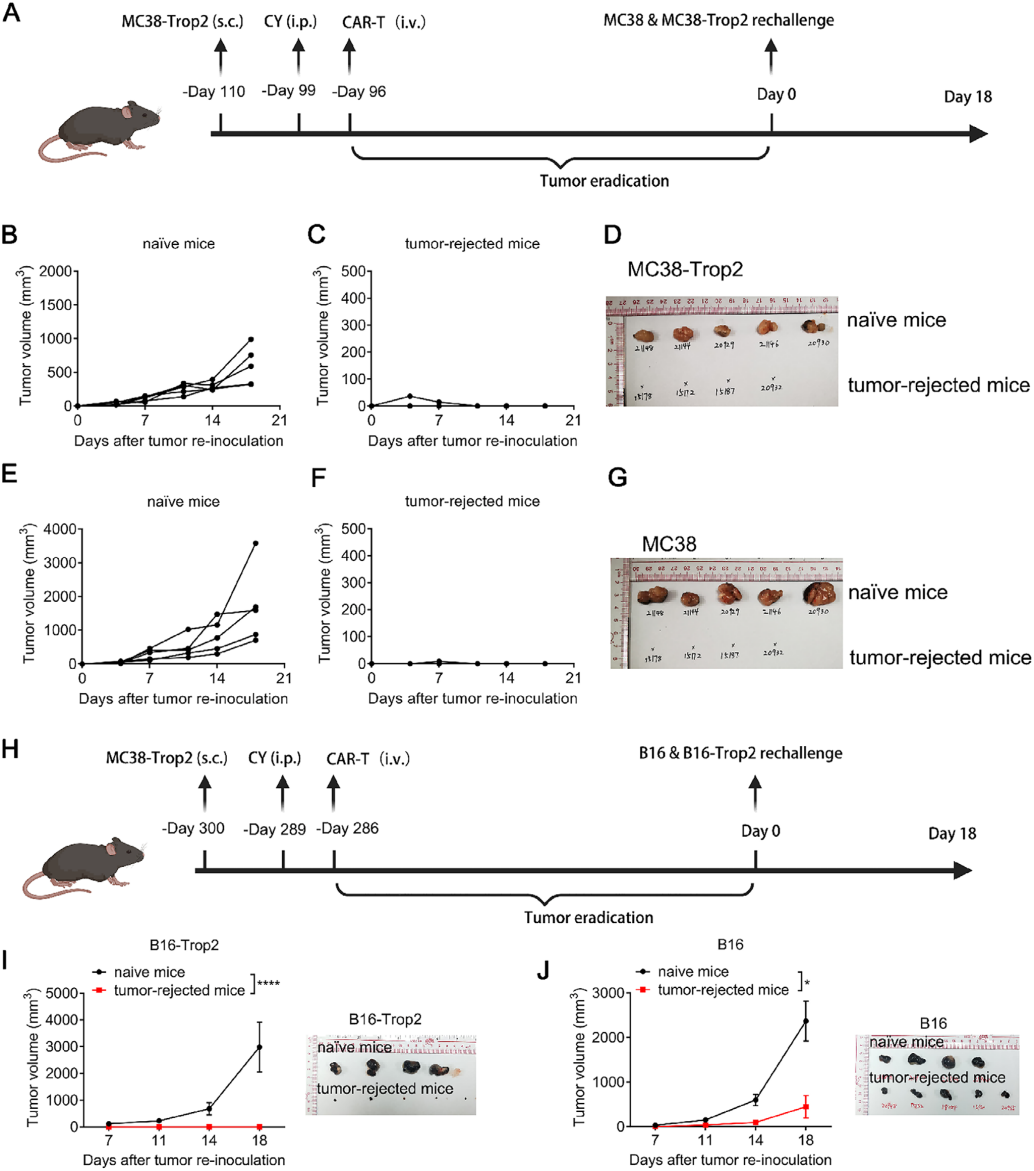
3P7‐CAR‐T cells prevent tumor recurrence. (A) The schematic diagram of MC38 / MC38‐Trop2 tumor re‐challenge assay. Mice were first inoculated with MC38‐Trop2, and treated with CAR‐T cells. At day 96 after CAR‐T cell infusion, long‐term surviving mice were re‐challenged with a second inoculation of MC38‐Trop2 cells on the right and parental MC38 cells on the left flanks (N = 4). As control, naïve C57 mice were inoculated with the tumors in the same paradigm (N = 5). (B) MC38‐Trop2 tumor growth in naïve mice. (C) MC38‐Trop2 tumor growth in 3P7‐CAR‐T treated, tumor rejected mice. (D) The image of MC38‐Trop2 tumors. (E) MC38 tumor growth in naïve mice. (F) MC38 tumor growth in 3P7‐CAR‐T tumor rejected mice. (G) The image of MC38 tumors. (H) The schematic diagram of B16 / B16‐Trop2 tumor re‐challenge assay. 286 days after CAR‐T infusion. The MC38‐Trop2 tumor‐rejected mice were re‐challenged with a second inoculation of tumor cells (N = 4 for naïve mice and N = 5 for tumor‐rejected mice). (I) The volumes of B16‐Trop2, and (J) B16 tumors were assessed. Data are presented as mean ± SEM. Statistical significance: *p<0.05, **p<0.01, ***p<0.001, ****p<0.0001. Two‐way ANOVA with Tukey's post‐test (I and J).

To further assess T cell memory induced by 3P7‐CAR‐T cells in vivo, we rechallenged MC38‐Trop2 tumor‐bearing mice achieving complete remission following 3P7‐CAR‐T therapy. Upon secondary challenge with tenfold higher tumor cells of either MC38‐Trop2 or MC38‐WT cells 110 days post tumor clearance, 3P7‐CAR‐T‐treated mice exhibited robust suppression of both tumor types (Figure 5A–G). This durable anti‐tumor efficacy was recapitulated in MC38‐CD19 models achieving complete remission after treatment with CD19‐targeted 3P7‐CAR‐T cells (Figure S6H–J). The Above results demonstrated that 3P7‐CAR‐T cells drive dual induction of antigen‐specific and endogenous T cell memory to control tumors.

We further validate the long‐term anti‐tumor efficacy of 3P7 CAR‐T cells in mice that had achieved complete remission from primary MC38‐Trop2 tumors following a 300‐day tumor‐free interval. Mice were separately inoculated with B16‐Trop2 tumor on the right and B16‐WT tumor on the left flank (Figure 5H). Strikingly, 3P7‐CAR‐T treated mice exhibited potent suppression of both B16‐Trop2 and B16‐WT tumors (Figure 5I,J). These results further confirmed the establishment of durable endogenous T cell memory following 3P7‐CAR‐T treatment.

Thus, the above results collectively indicated that CCL3 synergizes with IL‐7 to drive both CAR‐T and endogenous T cells toward Tcm differentiation and residency, thereby potentiating anti‐tumor effector functions.

### 2.6 3P7‐CAR‐T Reprogrammes Tumor Immune Microenvironment

**FIGURE 6:**
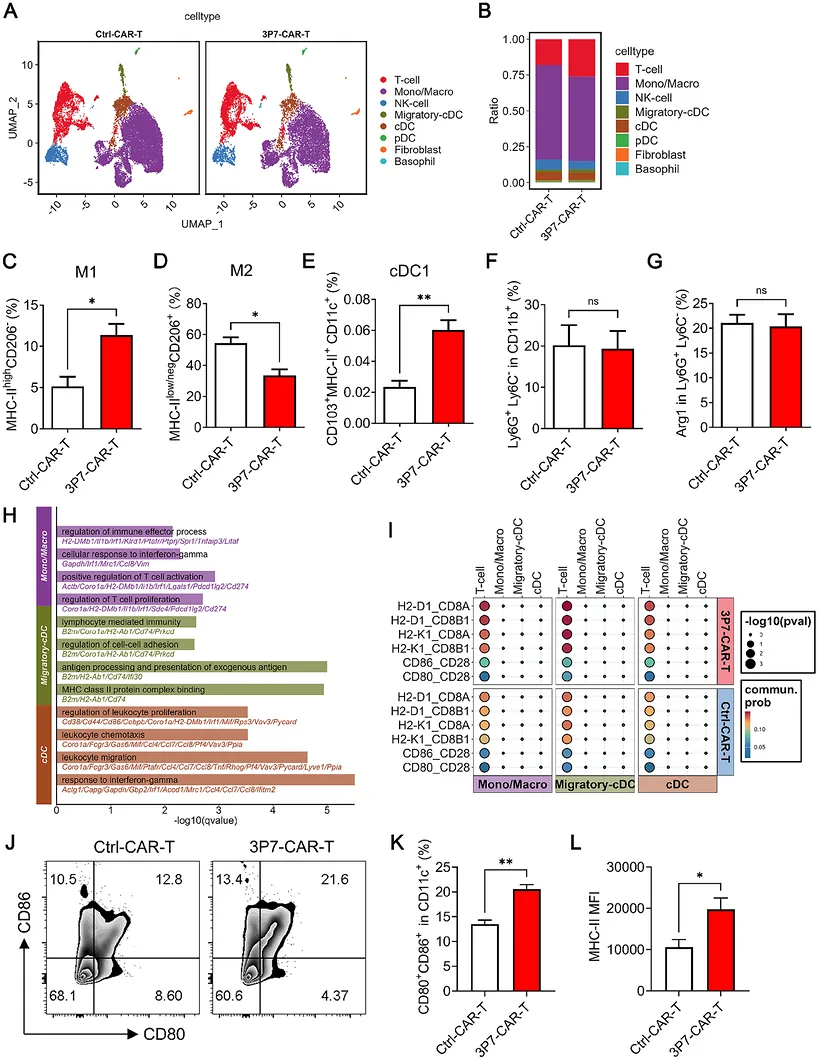
3P7‐CAR‐T reprograms the tumor immune microenvironment. (A) CD45^+^ cells were sorted from MC38‐Trop2 tumors treated with Ctrl‐CAR‐T and 3P7‐CAR‐T (N = 3/group), single cell RNA sequencing (scRNA‐seq) was performed. UMAP analysis of scRNA‐seq without T cell populations were performed, and 8 unique populations were identified. (B) The proportional changes of the subcluster immune cells in Ctrl‐CAR‐T and 3P7‐CAR‐T treated tumors. (C) The proportion of M1‐like (MHCII^high^CD206^−^) and (D) M2‐like (CD206^+^MHC‐II^low/neg^) cells in CD45^+^CD3^−^CD11b^+^F4/80^+^ TAMs were detected by flow cytometry in tumor treated with CAR‐T cells (N = 3/group). (E) Flow cytometric analysis of CD103^+^MHCII^+^CD11c^+^ DC1 in CD45^+^ cells in tumor treated with CAR‐T cells (N = 3/group). (F) The proportion of Ly‐6G^+^/Ly‐6C^−^ MDSCs in CD45^+^CD11b^+^ cells in tumors (N = 5/group). (G) The proportion of Arg1^+^ cells in Ly‐6G^+^/Ly‐6C^−^ MDSCs in tumors (N = 5/group). (H) GSVA enrichment analysis of Macrophages, Migratory DC and cDC cells from MC38‐Trop2 tumors treated with Ctrl‐CAR‐T and 3P7‐CAR‐T cells. (I) Cell chat and signaling analysis among diverse immune cells by using scRNA‐seq. (J) Flow cytometric analysis of the proportion of DCs expressing CD80 and CD86 in TDLN of MC38‐Trop2 tumor‐bearing mice. (K) The histogram shows the percentage of CD80^+^/CD86^+^ cells in TDLN (N = 3/group). (L) The levels of MHC‐II in CD45^+^CD11c^+^ cells in TDLN (N = 3/group). All data are presented as mean ± SEM. Statistical significance: *p<0.05, **p<0.01, ***p<0.001, ****p<0.0001. Student's *t* test (C–G, K, and L).

The immunosuppressive TME represents a critical determinant of functional impairment in tumor‐infiltrating T cells. Bioinformatic analysis identified CCL3 as a pivotal factor in remodeling the immune landscape by promoting infiltration of immunostimulatory myeloid populations, including M1 macrophages and CD103^+^ DCs (Figure S1K–M). To evaluate the capacity of 3P7‐CAR‐T cells to reprogram the TME in solid tumors, we analyzed scRNA‐seq data and revealed significant polarization of monocyte/macrophage within 3P7‐CAR‐T treated tumors, concomitant with marked enrichment of DC subsets, particularly migratory DCs (Figure 6A,B, and Figure S7A–C). Further flow cytometric results demonstrated an increase in immunostimulatory M1 macrophages (Figure 6C), but a suppression of immunosuppressive M2 macrophages (Figure 6D). Notably, CD103^+^ migratory DCs (DC1) associated with T cell activation were robustly expanded (Figure 6E,F), while Ly6G^+^ myeloid‐derived suppressor cells (MDSCs) and their expression of Arg‐1 remained unchanged (Figure 6F,G). These findings indicate that 3P7‐CAR‐T therapy drives preferential infiltration of antigen‐presenting cells (APCs) such as M1 macrophages and DC1, shifting the tumor microenvironment toward immune activation.

Besides, to delineate which factors are critical for memory phenotype formation, CD103^+^ dendritic cell induction, and macrophage reprogramming. We performed comparative experiments in MC38‐Trop2 tumor‐bearing mice treated with CAR‐T cells secreting IL‐7 alone, CCL3 alone, or both factors, and analyzed their effects on T cell memory phenotype formation, CD103^+^ dendritic cell induction, and macrophage reprogramming. The results showed that IL‐7 alone promoted T cell infiltration and memory differentiation, while CCL3 alone enhanced cDC1 infiltration and M1 polarization of macrophages (Figure S7D–I). In contrast, 3P7‐CAR‐T cells co‐expressing CCL3 and IL‐7 exhibited synergistic effects, concurrently promoting memory T cell formation, CD103^+^ dendritic cell induction, and M1 macrophage reprogramming within the TME.

Moreover, functional profiling of tumor‐associated macrophages (TAMs) and DCs revealed significant enrichment of antigen presentation and T cell activation pathways in 3P7‐CAR‐T treated tumors (Figure 6H). Ligand‐receptor pair analysis further identified enhanced MHC‐TCR interactions and CD80/86‐CD28 co‐stimulatory signaling within 3P7‐CAR‐T groups, corroborating the improved APCs‐T cell crosstalk (Figure 6I). We further confirmed the upregulated MHC‐II and maturation of DCs from 3P7‐CAR‐T‐treated mice (Figure 6J–L). Collectively, these data demonstrate that 3P7‐CAR‐T therapy effectively reprogrammed the TME, potentiating antigen presentation and T cell priming to achieve tumor clearance.

## 3 Discussion

The clinical success of CAR‐T cell therapy hinges on the sustained cytotoxic activity and intratumoral persistence of engineered T cells [20, 21]. While this modality has revolutionized hematologic malignancy treatment, its efficacy in solid tumors remains constrained, mainly by the suppressive TME, where inadequate chemotactic and suppressive signaling converge to limit therapeutic potential [22, 23]. Our findings revealed that strategic modulation of chemokine networks, specifically, the synergistic expression of CCL3 plus IL‐7 in CAR‐T cells, overcomes these barriers by enhancing both T cell trafficking and functional longevity, while concurrently reprogramming suppressive TME.

Chemokines are pivotal coordinators of T cell recruitment and activation [24, 25], yet their suboptimal expression in solid tumors creates a critical bottleneck for adoptive cell therapies [26]. Consequently, the genetic modification of therapeutic cells to overexpress chemokines has emerged as a novel strategy to improve cellular therapies for cancer [27]. However, chemokines constitute a large family comprising nearly 50 members. Despite the critical roles in regulating T cell responses, the comparative analysis of anti‐tumor chemokines remains poor. Here, we systematically compared the association between chemokines and effector T cell functionality in tumors by defining a T cell killing gene signature (TCKsig). We found CCL3 was one of the most relevant chemokines with TCKsig using published scRNA‐seq data, and CCL3 was strongly positively correlated with T cell cytotoxicity across TCGA solid tumors.

Prior efforts to augment chemokine signaling, such as engineering CAR‐T cells with CCL19, have potentiated anti‐tumor efficacy but remain considerably contingent on lymphodepletion pretreatment [11]. However, 3P7‐CAR‐T cells mediated robust tumor control independent of such pretreatment (Figure 3E), highlighting the therapeutic potential of 3P7‐CAR‐T cells for chemotherapy intolerant patients. We also constructed CCL3‐ and CCL19‐ CAR‐T cells co‐expressing IL‐7 (termed 3P7‐ and 19P7‐CAR‐T, respectively). In CT26 tumor models, a representative syngeneic system characterized by a highly immunosuppressive microenvironment and microsatellite stable (MSS) status [28], 3P7‐CAR‐T cells showed superior therapeutic efficacy (Figure S8). These findings suggest a potentially difference among chemokines in augmenting CAR‐T cell performance, wherein integrating chemokines that optimally enhance T cell cytotoxicity may significantly improve outcomes in solid tumor therapies.

IL‐2, IL‐7, IL‐10, IL‐12, and IL‐15 have all been explored as payloads for enhancing CAR‐T cell function, yet they exert distinct effects on T cell differentiation and persistence [29, 30]. IL‐2 and IL‐12 promote robust proliferation but are also associated with terminal differentiation and exhaustion [31, 32]. IL‐2, in particular, can drive effector differentiation at the expense of memory formation [33, 34]. IL‐12 potently enhances effector function and antitumor activity but may increase the risk of excessive inflammation and exhaustion [30]. Recent studies have shown that IL‐10 preserves stemness and metabolic fitness, although its effects are context dependent [35]. IL‐15 engineering in CAR‐T therapy has demonstrated promising antitumor efficacy against solid tumors in recent clinical trials [36]. However, it also leads to a higher incidence of CRS toxicity [36]. In contrast, IL‐7 is well established for its critical role in supporting T cell survival, promoting a central memory phenotype, and maintaining stem cell memory populations, features that are closely linked to sustained antitumor activity and long‐term persistence [37, 38]. Indeed, in the present study, we found that IL‐7 in combination with CCL3 synergistically promoted the differentiation of T cells toward a memory phenotype. Accordingly, 3P7 CAR‐T cells induced the formation of substantial endogenous memory T cells within the tumor and elicited durable antitumor responses. Importantly, the observation that mice achieving complete remission from MC38‐Trop2 tumors exhibited potent suppression of B16‐WT tumor rechallenge supports the notion that 3P7 CAR‐T therapy also induces endogenous T cell responses against shared tumor antigens, such as p15E [19, 39, 40], thereby providing cross‐protection against antigenically distinct tumors.

Tumor recurrence represents another key challenge that CAR‐T therapy must address in clinical applications, primarily due to tumor heterogeneity and antigen escape [41, 42]. Consequently, activation of endogenous T cell immunity and establishment of immunological memory emerge as pivotal mechanisms for sustaining durable remissions [43, 44]. Memory T cells exhibit heterogeneity in phenotype, effector function, and homing capability [45]. Central memory T cells (Tcm) express CD44 and CD62L, retaining naive T cell migration properties to home to secondary lymphoid organs. As antigen‐experienced cells with long‐term persistence, Tcm provide systemic protection and generate secondary effector cells [46, 47]. In contrast, Trm do not recirculate and permanently reside at barrier sites, forming the dominant memory population [48]. Both long‐lived memory CAR‐T cells are critical for improving tumor clearance and long‐term protection [49]. In this study, we demonstrated that 3P7‐CAR elicits synergistic signaling to modulate Trm transcriptor RUNX3, which both enhances memory and residency‐associated phenotypes by upregulating CD69 and CD103 endogenously in CAR‐T cells and exogenously in tumor‐infiltrating lymphocytes (TILs). Such orchestrated regulation effectively counteracts tumor heterogeneity‐mediated relapse. However, the role of CCL3 in Trm differentiation remains unclear. While IL‐7 is critical for memory T cell homeostasis, this conclusion is largely derived from circulating subsets rather than Trm [50]. And the mechanism whereby CCL3 and IL‐7 synergistically promote Trm differentiation requires further investigation.

Over the past decades, immunotherapy has led to a paradigm shift in cancer treatment by targeting the suppressive TME [4, 51]. This has been achieved through a variety of strategies, including enhancing the activity of DCs [52], antagonizing inhibitory TAMs [53], and targeting regulatory T cells (Tregs) [54]. However, the efficacy of such approaches targeting TME remains suboptimal. Through the administration of 3P7 CAR‐T cells, we observed that CCL3 plus IL‐7 synergistically reprogrammed the TME, facilitating the infiltration and maturation of pro‐inflammatory and APCs such as M1 macrophages and CD103^+^ DCs. This process further strengthened antigen‐presenting capacity and interaction for T cell responses.

In summary, this study demonstrated that CCL3 plus IL‐7 synergistically enhanced CAR‐T cell infiltration and persistence in solid tumors, ultimately leading to improved tumor eradication. This synergistic effect exhibited an intrinsic mechanism irrespective of tumor types, CAR‐T administration routes, target specificities, or intracellular signaling domains. Mechanistically, CCL3 plus IL‐7 upregulated RUNX3 in T cells, promoting CAR‐T cell memory formation and enhancing TILs memory differentiation. Furthermore, scRNA‐seq and tumoral analyses revealed that 3P7‐CAR‐T therapy reshaped myeloid populations, increasing M1 macrophages and DCs maturation, thereby amplifying antigen presentation and secondary T cell priming. This bidirectional crosstalk between adoptively transferred CAR‐T cells and endogenous immune cells establishes a self‐reinforcing anti‐tumor loop, providing a robust therapeutic strategy for CAR‐T therapy as well as mitigating relapse risks posed by tumor heterogeneity.

## 4 Materials and Methods

### 4.1 Retroviral Vectors Design and Construction

The human Trop2‐specific murine CAR (T2‐m28z) was generated by linking anti‐human Trop2 single‐chain fragment variable (scFv) to the mouse CD8α hinge domain, mouse CD28 transmembrane, and the cytoplasmic domain, and the intracellular signaling domain of mouse CD3ζ. CAR genes were then cloned into the retroviral vector MIGR1 which was used as previously described [15]. To construct a vector encoding CCL3 and IL‐7 in murine CAR, the CCL3 and IL‐7 genes were inserted into the *BamHI* and *ClaI* restriction enzyme sites of the MIGR1 retroviral vector, and 2A self‐cleavable peptide from porcineteschovirus‐1 polyprotein was inserted between T2‐m28z and 3P7 CAR‐T cell generation.

Retrovirus production and CAR‐T cells transduction were conducted as we described previously [14, 15]. Briefly, 293T cells were transfected with the CAR‐expressing retrovirus plasmid and pCL‐Eco packaging plasmid by using the calcium phosphate method. The culture supernatants were harvested and frozen for gene transduction after 48 h. Mouse T cells isolated from wild‐type C57BL/6 or BALB/c female mouse splenocytes were purified with a mouse CD3^+^ T cell negative enrichment kit (BD, USA). T cells were activated in 0.2 mg/mL rabbit anti‐hamster (A18897; Invitrogen) precoated 24‐well plate, cultured in RPMI 1640 (Invitrogen) containing 2 mm L‐glutamine, 50 mm 2‐ME, 100 U/mL penicillin, 100 mg/mL streptomycin and 10% fetal bovine serum (FBS) supplemented with 0.25 µg/mL hamster anti‐CD3 (145–2C11; eBioscience; 16‐0031‐85), 1 µg/mL hamster anti‐CD28 (37.51; eBioscience; 16‐0281‐85), and 10 ng/mL murine IL‐2 (R&D) for 24 h. The activated T cells were then infected with retroviral supernatants in the presence of RetroNectin (Takara Bio) and transduction efficacy was determined in GFP^+^ cells with flow cytometry analysis.

### 4.2 Cell Lines

293T‐17(ATCC, RRID: CRL‐11268) and COLO‐205 cells(ATCC, RRID: CCL‐222) were obtained from ATCC and maintained in conditioned high‐glucose DMEM (Gibco, Invitrogen, Carlsbad, California, USA) supplemented with 10% Fetus Bovine Serum (FBS) and 100 U/mL penicillin/streptomycin (Invitrogen). MC38 (ATCC, RRID: CRL‐2640), B16‐F10 (ATCC, RRID: CRL‐6475), LLC (ATCC, RRID: CRL‐1642), and 4T1 (ATCC, RRID: CRL‐2539) cells were transduced with recombinant retroviruses carrying Trop2‐IRES‐GFP moiety to establish MC38‐Trop2, B16‐F10‐Trop2, LLC‐Trop2, and 4T1‐Trop2 cells, followed by GFP high sorting (BD FACSAria III). Tumor cells were cultured in RPMI 1640 (Invitrogen) containing 10% FBS and 100 U/mL penicillin/streptomycin. All cell lines were maintained in a humidified atmosphere at 37°C and 5% CO_2_.

### 4.3 Flow Cytometry

The following antibodies were used for flow cytometry analysis: PE conjugated anti‐mouse TCR (clone H57‐597, Invitrogen), SB600 conjugated anti‐mouse CD8 (clone 53–6.7, Invitrogen), APC‐Cyanine7 conjugated anti‐mouse CD45 (clone 30‐F11, Invitrogen), PerCP‐Cyanine5.5 conjugated anti‐mouse CD4 (clone RM4‐5, Invitrogen), APC conjugated anti‐mouse CD8 (clone 53–6.7, Invitrogen), PE‐Cyanine7 conjugated anti‐mouse CD3 (clone 145–2 C11, Invitrogen), APC conjugated anti‐mouse PD‐1 (clone J43, Invitrogen), PerCP‐Cyanine5.5 conjugated anti‐mouse Tim3 (clone RMT3‐23, Invitrogen), PE‐Cyanine7 conjugated anti‐mouse Lag‐3 (clone C9B7W, Invitrogen), Pacific Blue conjugated anti‐mouse CD4 (clone RM4‐5, Invitrogen), PerCP‐Cyanine5.5 conjugated anti‐mouse CD11b (clone M1/70, Invitrogen), PE conjugated anti‐mouse F4/80 (clone BM8, Invitrogen), Pacific Blue conjugated anti‐mouse IFN‐γ (clone XMG1.2, Invitrogen), PE conjugated anti‐mouse TNF‐α (clone MP6‐XT22, Invitrogen), PE‐Cyanine7 conjugated anti‐mouse Granzyme‐B (clone NGZB, Invitrogen), PerCP‐Cyanine5.5 conjugated anti‐mouse CD11c (clone N418, TONBO Biosciences), PerCP‐Cyanine5.5 conjugated anti‐mouse CD44 (clone IM7, Invitrogen), APC‐eFlour780 conjugated anti‐mouse CD62L (clone MEL‐14, Invitrogen), PE‐Cyanine7 conjugated anti‐mouse CD206 (clone MR6F3, Invitrogen), APC conjugated anti‐mouse MHCII (clone AF6‐88.5.5.3, Invitrogen), APC conjugated anti‐mouse Ly6C (clone AL‐21, BD Biosciences), Percp‐Cy5.5 conjugated anti‐mouse Ly6G (clone 1A8, BD Biosciences), BV421 conjugated anti‐mouse Gr‐1 (clone RB6‐8C5, BD Biosciences), PE conjugated anti‐mouse Arg1 (clone A1exF5, Invitrogen), PE conjugated anti‐mouse H‐2K^b^ MuLV p15E Tetramer (Cat: TS‐M507‐1, MBL), PE‐conjugated anti‐mouse CD103 (clone 2E7, Invitrogen), APC‐conjugated anti‐mouse‐CD69 (clone H1.2F3, Invitrogen), FITC conjugated anti‐mouse CD80 (clone 16‐10A1, Invitrogen), PE conjugated anti‐mouse‐CD86 (clone GL1, Invitrogen), live or dead cells were defined by Fixable Viability Stain 700 (564997, BD), and PE conjugated anti‐human Trop2(clone MR54, Invitrogen). FACS analysis was performed with flow cytometry (LSR Fortessa, BD) and FlowJo software (FlowJo_V10, BD). An Annexin V‐PE Apoptosis Detection Kit (BD Pharmingen) was used to evaluate apoptosis levels according to the manufacturer's guidelines.

### 4.4 ELISA

Concentrations of CCL3 and IL‐7 in the supernatants of several CAR‐T cells cultured alone or after co‐culture with MC38‐Trop2 cells for 24 h were determined using a commercial mouse CCL3 enzyme‐linked immunosorbent assay (ELISA) kit (Cat: ELM‐MIP1a, RayBioech), and a mouse IL‐7 ELISA kit (Cat: ELM‐IL7, RayBioech) according to the manufacturer's instructions.

### 4.5 In Vitro Cytotoxicity and Activation Assays of CAR‐T Cells

The cytotoxic activity of the CAR‐T cells was evaluated using the CytoTox 96 Non‐Radioactive Cytotoxicity Assay (G1781, Promega) following manufacturer's guidelines. The lactate dehydrogenase (LDH) release was evaluated after 8 h in the supernatant with effector‐to‐target ratios of 1:1, 2:1, and 5:1.

For intracellular cytokine detection. CAR‐T cells were cocultured with tumor cells at a 1:2 ratio, with 5 × 10^5^ T cells seeded in 24‐well flat‐bottom tissue culture plates for 96 h. Then brefeldin A inhibitor was added and cells were harvested 4 h later and stained with anti‐mouse CD3 antibody. The cells were then fixed, permeabilized, and stained with anti‐mouse IFN‐γ, TNF‐α and GranzymeB according to the manufacturer's guidelines (88‐8824‐00, eBioscience Intracellular Fixation and Permeabilization Buffer Set). For the tests of T cell apoptosis in vitro, 5 × 10^5^ CAR‐T cells were stimulated with 1 × 10^6^ MC38‐Trop2 cells in 12‐well tissue culture plates. After 96 h, CAR‐T cells were harvested to assess the level of apoptosis, expression of memory‐related proteins, and exhaustion markers.

### 4.6 Animal Models

To assess the anti‐tumor effects of CAR‐T cells in the established MC38‐Trop2^+^ mice tumor models, 6–8 weeks old female C57BL/6 mice were inoculated subcutaneously (*s.c*.) with 5 × 10^5^ tumor cells in the right flank (0 d), and then cyclophosphamide (CY: 80 mg/kg, Sigma) was administered intraperitoneally (*i.p*.) 11 d after tumor inoculation. On day 14, 1 × 10^6^ CAR‐T cells were injected intravenously. In some experiments, C57BL/6 mice were inoculated s.c. with 5 × 10^5^ MC38‐Trop2^+^ tumor cells on 0 d. At 7 d, 1 × 10^6^ CAR‐T cells were injected intravenously without CY pretreatment. In the 4T1‐Trop2^+^ model, BALB/c mice were inoculated (*s.c*.) with 5 × 10^5^ tumor cells in the right flank (0 d), and then injected intravenously with 1 × 10^6^ CAR‐T cells at 10 d with CY pretreatment. The mouse lung cancer model was inoculated (*s.c*.) with 1 × 10^6^ LLC‐Trop2 + cells (0 d), and then injected intravenously with 1 × 10^6^ CAR‐T cells at 14 d with CY pretreatment. Metastatic tumor models were established by intravenous injection of 1 × 10^6^ MC38‐Trop2 tumor cells (*i.v*.), followed by CAR‐T cell infusion at day 14 post‐injection. NCG mice (NOD/ShiLtJGpt‐Prkdc^em26Cd52^Il2rg^em26Cd22^/Gpt, Nanjing GemPharmatech) were inoculated with Colo‐205 cells (*s.c*.) followed by 1 × 10^6^ transduced human CAR T cells infusion at day 7. In the model using a mixture of MC38 and MC38‐Trop2 cells, C57BL/6 mice were inoculated subcutaneously with 5 × 10^5^ MC38 and 5 × 10^5^ MC38‐Trop2 tumor cells on the right flank on day 0, and 1 × 10^6^ CAR‐T cells were injected *i.v*. into the mice at day 10 with CY pretreatment. In all experiments, tumor growth was measured by digital calipers, and tumor volumes were calculated on the basis of volume = 1/2 × length × (width)^2^. For TME analysis, C57 mice were inoculated with 5 × 10^5^ MC38‐Trop2 tumor cells (*s.c*.). At 14 d, 1 × 10^6^ CAR‐T cells were injected intravenously without CY pretreatment. On day 21, spleen cells and tumor‐infiltrating lymphocytes were harvested to assess the ratio and kinetics of the CAR‐T cells. The animal study was approved by the Institutional Animal Care and Use Committee (IACUC) of Guangzhou National Laboratory under approval number GZLAB‐AUCP‐2024‐04‐A01. Human peripheral blood from healthy donors used for the isolation of T cells was purchased from Hycells (Cat: hPBLP001, Shanghai, China).

### 4.7 scRNA‐Seq Data Analysis

All raw sequencing data generated in this study have been submitted to the Gene Expression Omnibus (GEO) under accession numbers GSE330826 (for scRNA‐seq and TCR‐seq), and GSE330502 (for RNA‐seq). The Cell Ranger Single‐Cell software suite (10x Genomics) was used to process the scRNA‐seq FASTQ files. For the datasets with matched TCR‐seq data, the vdj command was used to generate a count matrix, which, after filtering, was used for downstream analyses. For single‐cell gene expression analysis, the filtered count matrices were processed using Seurat (v.4.4.0) in R (v.4.2.3), where Ctrl and 3P7‐CAR‐T samples, along with their corresponding CD45^+^ immune cell populations were integrated into a unified Seurat object to ensure consistent processing across all datasets. We performed 34,845 high‐quality cells and analysis proceeded with the selection of 2000 highly variable genes followed by principal component analysis (PCA).

The miloR R package (v. 2.3.1) was used to assess differential abundance within overlapping cell neighborhoods, ranging in size from 50 to 200 cells each. For each group (Ctrl‐CAR‐T and 3P7‐CAR‐T), the expression matrix was subset to include only the top 3,000 most variable genes within cells from control and treated Chimeroids, PCA was performed on that submatrix and the result was passed into a Milo object for further analysis.

### 4.8 TCR Data Analysis and Visualization

For TCR‐seq, filtered contig annotation matrices from the Cell Ranger output were loaded into R. Annotation and quantification of TCR clonotypes were processed with the scRepertoire package (1.8.0). Clonal frequencies in CD8^+^T cells (Cd3d^+^ Cd8a^+^) were categorized as follows: single (1), small (2–5), medium (6–10), large (11–50), and hyper (51–500). Clonal expansion bar plots were generated using the ggplot2 R package. The clonotypic information was integrated into the Seurat object using the combineExpression function, with the cloneTypes variable set to cloneTypes = c ((Single = 1, Small = 5, Medium = 10, Large = 50, and Hyperexpanded = 500). The cell frequencies of different clonetype sizes were further plotted as a stacked bar plot using the ggplot2 R package. Cells were considered clonally expanded if the clonotype size was greater than 1 and non‐expanded if the clonotype size was equal to 1.

### 4.9 TCGA Analysis

The results shown here are in whole or part based on data generated by the TCGA Research Network (http://cancergenome.nih.gov/). Normalized and batch‐adjusted RNA‐seq data were obtained from the UCSC Xenabrowser (https://xenabrowser.net/datapages/). Additional publicly available RNAseq datasets used in this study include E‐MTAB‐6149, GSE118056, and E‐MTAB‐14590, Normalized gene expression data and associated sample metadata were extracted directly from Gene Expression Omnibus. Specifically, to assess whether expression of CCL3 is associated with the survival outcome of patients with cancer, we assigned patients with cutaneous melanoma from the TCGA cohort to high or low groups based on the value of expression of CCL3 and performed overall survival analysis. The survival results were displayed using Kaplan‐Meier curves. *p*‐value = 0.05 was used as the threshold of statistical significance.

### 4.10 Statistical Analysis

Data were analyzed using Prism (V.10.0, GraphPad) software and the results were presented as mean ± SEM where indicated. The comparison of two groups was performed using Student's two‐tailed *t*‐test, while comparisons among more than three groups were analyzed using one‐way analysis of variance (ANOVA) with Tukey's post‐test. Tumor growth curve comparisons were analyzed by Two‐way ANOVA with Tukey's post‐test. Survival curves were calculated by implementing a Log‐rank (Mantel‐Cox) test. Correlation analysis was performed using Spearman's rank correlation coefficient. ^*^p<0.05, ^**^p<0.01, ^***^p<0.001, ^****^p<0.0001 were considered significant.

## Author Contributions

Z.H., H.C., and Y.B. proposed the research concept and designed the experiments; H.C., H.G., and X.L. performed all the experiments and provided essential experimental resources; Y.Z. and Y.K. assisted with the bioinformatic RNA‐seq and single‐cell RNA‐seq data analysis. Y.Z., X. Xu., and X. X. generated CAR retroviral vectors. F.W. and S.Y. performed H&E staining. Z.L. and B.Y. provided support for the animal experiment. Y.B., H.C., and Y.K. analyzed the data; Y.B., H.C. wrote the manuscript.

## Funding

This work was supported by funds from the Major Project of Guangzhou National Laboratory (GZNL2024A02004 and GZNL2023A02009) to Y. B., the National Natural Science Foundation of China (32370971) to Y. B., the Young Talent Program of China (HJJH22007) to Y. B. and the National Natural Science Foundation of China (32500797) to H.C. Additionally, the contributions of OA in this article were financially supported by GZNL2024A02004 and NSFC‐32500797. The funding source was not involved in any aspect of this research, including the study design, data collection and analysis, manuscript preparation, or the decision to submit the paper for publication.

## Ethics Statement

All mouse experiments were approved by the Institutional Animal Care and Use Committee (IACUC) of Guangzhou National Laboratory, GZLAB‐AUCP‐ 2024‐04‐A01. All tumor volumes were strictly maintained below the Ethics Committee's maximum allowable volume of 2000 mm^3^. Mice were euthanized once tumor volume reached or surpassed this limit.

## Consent

All the authors have read and approved the final manuscript for publication.

## Conflicts of Interest

A patent related to the methodology presented in this article has been granted (Patent No. ZL 2021 1 0867752. 8) to Sun Yat‐Sen University, with H. C., and Z.H. listed as inventors. The authors declare no other competing interests.

## Supporting information




**Supporting File 1**: advs75993‐sup‐0001‐SuppMat.docx.

**Supporting File 2**: advs75993‐sup‐0002‐Data.zip.

## Data Availability

The data that support the findings of this study are available from the corresponding author upon reasonable request.

## References

[1] J. N. Brudno , M. V. Maus , and C. S. Hinrichs , “CAR T Cells and T‐Cell Therapies for Cancer,” Jama 332, no. 22 (2024): 1924–1935, 10.1001/jama.2024.19462. 39495525 PMC11808657

[2] N. N. Shah , B. D. Johnson , D. Schneider , et al., “Bispecific Anti‐CD20, Anti‐CD19 CAR T Cells for Relapsed B Cell Malignancies: A Phase 1 Dose Escalation and Expansion Trial,” Nature medicine 26, no. 10 (2020): 1569–1575, 10.1038/s41591-020-1081-3. 33020647

[3] Y. Liu , C. Peng , F. Ahad , S. A. Ali Zaidi , T. A. Muluh , and Q. Fu , “Advanced Strategies of CAR‐T Cell Therapy in Solid Tumors and Hematological Malignancies,” Recent Patents on Anti‐Cancer Drug Discovery 19, no. 5 (2024): 557–572, 10.2174/0115748928277331231218115402. 38213150

[4] R. Polak , E. T. Zhang , and C. J. Kuo , “Cancer Organoids 2.0: Modelling the Complexity of the Tumour Immune Microenvironment,” Nature reviews Cancer 24, no. 8 (2024): 523–539, 10.1038/s41568-024-00706-6. 38977835

[5] J. J. Peng , L. Wang , Z. Li , C. L. Ku , and P. C. Ho , “Metabolic Challenges and Interventions in CAR T Cell Therapy,” Science immunology 8, no. 82 (2023): abq3016, 10.1126/sciimmunol.abq3016. 37058548

[6] T. R. Mempel , J. K. Lill , and L. M. Altenburger , “How Chemokines Organize the Tumour Microenvironment,” Nature reviews Cancer 24, no. 1 (2024): 28–50, 10.1038/s41568-023-00635-w. 38066335 PMC11480775

[7] D. Dangaj , M. Bruand , A. J. Grimm , et al., “Cooperation Between Constitutive and Inducible Chemokines Enables T Cell Engraftment and Immune Attack in Solid Tumors,” Cancer Cell 35, no. 6 (2019): 885–900.e10, 10.1016/j.ccell.2019.05.004. 31185212 PMC6961655

[8] R. Zhang , L. Tian , L.‐J. Chen , et al., “Combination of MIG (CXCL9) Chemokine Gene Therapy With Low‐dose Cisplatin Improves Therapeutic Efficacy Against Murine Carcinoma,” Gene therapy 13, no. 17 (2006): 1263–1271, 10.1038/sj.gt.3302756. 16672984

[9] X. Wang , X. L. Lu , H. Y. Zhao , F. C. Zhang , and X. B. Jiang , “A Novel Recombinant Protein of IP10‐EGFRvIIIscFv and CD^8+^ cytotoxic T Lymphocytes Synergistically Inhibits the Growth of Implanted Glioma in Mice,” Cancer Immunology, Immunotherapy 62, no. 7 (2013): 1261–1272, 10.1007/s00262-013-1426-6. 23640602 PMC11029612

[10] Z. Liu , R. Ravindranathan , J. Li , P. Kalinski , Z. S. Guo , and D. L. Bartlett , “CXCL11‐Armed Oncolytic Poxvirus Elicits Potent Antitumor Immunity and Shows Enhanced Therapeutic Efficacy,” Oncoimmunology 5, no. 3 (2016): 1091554, 10.1080/2162402X.2015.1091554. PMC4839379 27141352

[11] K. Adachi , Y. Kano , T. Nagai , N. Okuyama , Y. Sakoda , and K. Tamada , “IL‐7 and CCL19 Expression in CAR‐T Cells Improves Immune Cell Infiltration and CAR‐T Cell Survival in the Tumor,” Nature biotechnology 36, no. 4 (2018): 346–351, 10.1038/nbt.4086. 29505028

[12] H. Luo , J. Su , and R. Sun , Coexpression of IL7 and CCL21 Increases Efficacy of CAR‐T Cells in Solid Tumors Without Requiring Preconditioned Lymphodepletion. Clinical Cancer Research: An Official Journal of the (American Association for Cancer Research, 2020). 10.1158/1078-0432.CCR-20-0777 32816947

[13] A. Zlotnik and O. Yoshie , “The Chemokine Superfamily Revisited,” Immunity 36, no. 5 (2012): 705–716, 10.1016/j.immuni.2012.05.008. 22633458 PMC3396424

[14] H. Chen , F. Wei , M. Yin , et al., “CD27 Enhances the Killing Effect of CAR T Cells Targeting Trophoblast Cell Surface Antigen 2 in the Treatment of Solid Tumors,” Cancer Immunology, Immunotherapy 70, no. 7 (2021): 2059–2071, 10.1007/s00262-020-02838-8. 33439295 PMC10992360

[15] H. Chen , Y. Yang , Y. Deng , et al., “Delivery of CD47 Blocker SIRPα‐Fc by CAR‐T Cells Enhances Antitumor Efficacy,” Journal for immunotherapy of cancer 10, no. 2 (2022): 003737, 10.1136/jitc-2021-003737. PMC8811602 35110357

[16] C. A. Arango‐Franco , M. Ogishi , S. Unger , et al., “IL‐7–Dependent and –Independent Lineages of IL‐7R–Dependent Human T Cells,” Journal of Clinical Investigation 134, no. 19 (2024): 180251, 10.1172/JCI180251. PMC11444196 39352394

[17] Y. Xu , X. Zhang , D. Xin , et al., “CD27‐Armored BCMA CAR T‐cell Therapy (CBG‐002) for Relapsed and Refractory Multiple Myeloma: A Phase I Clinical Trial,” Cancer immunology research 13, no. 1 (2025): 23–34, 10.1158/2326-6066.CIR-24-0051. 39432745

[18] N. Prokhnevska , M. A. Cardenas , R. M. Valanparambil , et al., “CD^8+^ T Cell Activation in Cancer Comprises an Initial Activation Phase in Lymph Nodes Followed by Effector Differentiation Within the Tumor,” Immunity 56, no. 1 (2023): 107–124.e5, 10.1016/j.immuni.2022.12.002. 36580918 PMC10266440

[19] X. Ye , J. C. Waite , A. Dhanik , et al., “Endogenous Retroviral Proteins Provide an Immunodominant but Not Requisite Antigen in a Murine Immunotherapy Tumor Model,” Oncoimmunology 9, no. 1 (2020): 1758602, 10.1080/2162402X.2020.1758602. 32923116 PMC7458611

[20] B. Du , J. Qin , B. Lin , J. Zhang , D. Li , and M. Liu , “CAR‐T Therapy in Solid Tumors,” Cancer Cell 43, no. 4 (2025): 665–679, 10.1016/j.ccell.2025.03.019. 40233718

[21] L. Liu , P. He , Y. Wang , et al., “Engineering Sonogenetic EchoBack‐CAR T Cells,” Cell 188, no. 10 (2025): 2621–2636.e20, 10.1016/j.cell.2025.02.035. 40179881 PMC12085297

[22] M. Hong , J. D. Clubb , and Y. Y. Chen , “Engineering CAR‐T Cells for Next‐Generation Cancer Therapy,” Cancer Cell 38, no. 4 (2020): 473–488, 10.1016/j.ccell.2020.07.005. 32735779

[23] J. Cheng , J. Yan , Y. Liu , et al., “Cancer‐Cell‐Derived Fumarate Suppresses the Anti‐Tumor Capacity of CD^8+^ T Cells in the Tumor Microenvironment,” Cell metabolism 35, no. 6 (2023): 961–978.e10, 10.1016/j.cmet.2023.04.017. 37178684

[24] R. J. Lim , R. Salehi‐Rad , L. M. Tran , et al., “CXCL9/10‐Engineered Dendritic Cells Promote T Cell Activation and Enhance Immune Checkpoint Blockade for Lung Cancer,” Cell reports Medicine 5, no. 4 (2024): 101479, 10.1016/j.xcrm.2024.101479. 38518770 PMC11031384

[25] M. Hong , A.‐L. Puaux , C. Huang , et al., “Chemotherapy Induces Intratumoral Expression of Chemokines in Cutaneous Melanoma, Favoring T‐Cell Infiltration and Tumor Control,” Cancer research 71, no. 22 (2011): 6997–7009, 10.1158/0008-5472.CAN-11-1466. 21948969

[26] S. Raza , S. Rajak , A. Tewari , et al., “Multifaceted Role of Chemokines in Solid Tumors: From Biology to Therapy,” Seminars in cancer biology 86 (2022): 1105–1121, 10.1016/j.semcancer.2021.12.011. PMC7613720 34979274

[27] R. Sun , H. Luo , J. Su , et al., “Olaparib Suppresses MDSC Recruitment via SDF1α/CXCR4 Axis to Improve the Anti‐Tumor Efficacy of CAR‐T Cells on Breast Cancer in Mice,” Molecular Therapy 29, no. 1 (2021): 60–74, 10.1016/j.ymthe.2020.09.034. 33010818 PMC7791086

[28] M. A. Ghonim , S. V. Ibba , A. F. Tarhuni , et al., “Targeting PARP‐1 With Metronomic Therapy Modulates MDSC Suppressive Function and Enhances anti‐PD‐1 Immunotherapy in Colon Cancer,” Journal for immunotherapy of cancer 9, no. 1 (2021): 001643, 10.1136/jitc-2020-001643. PMC7839867 33495297

[29] M. Bell and S. Gottschalk , “Engineered Cytokine Signaling to Improve CAR T Cell Effector Function,” Frontiers in immunology 12 (2021): 684642, 10.3389/fimmu.2021.684642. 34177932 PMC8220823

[30] C. Dong , “Cytokine Regulation and Function in T Cells,” Annual review of immunology 39 (2021): 51–76, 10.1146/annurev-immunol-061020-053702. 33428453

[31] N. K. Mehta , K. Rakhra , K. A. Meetze , et al., “CLN‐617 Retains IL2 and IL12 in Injected Tumors to Drive Robust and Systemic Immune‐Mediated Antitumor Activity,” Cancer immunology research 12, no. 8 (2024): 1022–1038, 10.1158/2326-6066.CIR-23-0636. 38842347 PMC11292205

[32] K. D. Moynihan , M. P. Kumar , H. Sultan , et al., “IL2 Targeted to CD^8+^ T Cells Promotes Robust Effector T‐Cell Responses and Potent Antitumor Immunity,” Cancer discovery 14, no. 7 (2024): 1206–1225, 10.1158/2159-8290.CD-23-1266. 38563906 PMC11215410

[33] T. Kaartinen , A. Luostarinen , P. Maliniemi , et al., “Low Interleukin‐2 Concentration Favors Generation of Early Memory T Cells Over Effector Phenotypes During Chimeric Antigen Receptor T‐Cell Expansion,” Cytotherapy 19, no. 6 (2017): 689–702, 10.1016/j.jcyt.2017.03.067. 28411126

[34] V. Kalia and S. Sarkar , “Regulation of Effector and Memory CD8 T Cell Differentiation by IL‐2—A Balancing Act,” Frontiers in immunology 9 (2018): 2987, 10.3389/fimmu.2018.02987. 30619342 PMC6306427

[35] T. Bedke , F. Muscate , S. Soukou , N. Gagliani , and S. Huber , “IL‐10‐Producing T Cells and Their Dual Functions,” Seminars in immunology 44 (2019): 101335, 10.1016/j.smim.2019.101335. 31734129

[36] D. Steffin , N. Ghatwai , A. Montalbano , et al., “Interleukin‐15‐Armoured GPC3 CAR T Cells for Patients With Solid Cancers,” Nature 637, no. 8047 (2025): 940–946, 10.1038/s41586-024-08261-8. 39604730 PMC12704925

[37] N. Cieri , B. Camisa , F. Cocchiarella , et al., “IL‐7 and IL‐15 Instruct the Generation of Human Memory Stem T Cells From Naive Precursors,” Blood 121, no. 4 (2013): 573–584, 10.1182/blood-2012-05-431718. 23160470

[38] Z.‐C. Ding , C. Liu , Y. Cao , et al., “IL‐7 Signaling Imparts Polyfunctionality and Stemness Potential to CD^4+^ T cells,” Oncoimmunology 5, no. 6 (2016): 1171445, 10.1080/2162402X.2016.1171445. PMC4938319 27471650

[39] H. D. White , D. A. Roeder , and W. R. Green , “An Immunodominant Kb‐Restricted Peptide From the p15E Transmembrane Protein of Endogenous Ecotropic Murine Leukemia Virus (MuLV) AKR623 That Restores Susceptibility of a Tumor Line to Anti‐AKR/Gross MuLV Cytotoxic T Lymphocytes,” Journal of virology 68, no. 2 (1994): 897–904, 10.1128/jvi.68.2.897-904.1994. 8289392 PMC236526

[40] K. Shitaoka , H. Hamana , H. Kishi , et al., “Identification of Tumoricidal TCRs From Tumor‐Infiltrating Lymphocytes by Single‐Cell Analysis,” Cancer immunology research 6, no. 4 (2018): 378–388, 10.1158/2326-6066.CIR-17-0489. 29475880

[41] M. Hamieh , A. Dobrin , A. Cabriolu , et al., “CAR T Cell Trogocytosis and Cooperative Killing Regulate Tumour Antigen Escape,” Nature 568, no. 7750 (2019): 112–116, 10.1038/s41586-019-1054-1. 30918399 PMC6707377

[42] R. L. Vincent , C. R. Gurbatri , F. Li , et al., “Probiotic‐Guided CAR‐T Cells for Solid Tumor Targeting,” Science 382, no. 6667 (2023): 211–218, 10.1126/science.add7034. 37824640 PMC10915968

[43] X. Si , M. Shao , X. Teng , et al., “Mitochondrial Isocitrate Dehydrogenase Impedes CAR T Cell Function by Restraining Antioxidant Metabolism and Histone Acetylation,” Cell metabolism 36, no. 1 (2024): 176–192.e10, 10.1016/j.cmet.2023.12.010. 38171332

[44] M. Wenes , A. Jaccard , T. Wyss , et al., “The Mitochondrial Pyruvate Carrier Regulates Memory T Cell Differentiation and Antitumor Function,” Cell metabolism 34, no. 5 (2022): 731–746.e9, 10.1016/j.cmet.2022.03.013. 35452600 PMC9116152

[45] L. Parga‐Vidal and K. van Gisbergen , “Area Under Immunosurveillance: Dedicated Roles of Memory CD8 T‐Cell Subsets,” Cold Spring Harbor perspectives in biology 12, no. 11 (2020): a037796, 10.1101/cshperspect.a037796. 32839203 PMC7605223

[46] X. Zhao , W. Hu , S. R. Park , et al., “The Transcriptional Cofactor Tle3 Reciprocally Controls Effector and Central Memory CD^8+^ T Cell Fates,” Nature Immunology 25, no. 2 (2024): 294–306, 10.1038/s41590-023-01720-w. 38238608 PMC10916363

[47] Q. Liu , Z. Sun , and L. Chen , “Memory T Cells: Strategies for Optimizing Tumor Immunotherapy,” Protein & cell 11, no. 8 (2020): 549–564, 10.1007/s13238-020-00707-9. 32221812 PMC7381543

[48] S. N. Mueller and L. K. Mackay , “Tissue‐Resident Memory T Cells: Local Specialists in Immune Defence,” Nature Reviews Immunology 16, no. 2 (2016): 79–89, 10.1038/nri.2015.3. 26688350

[49] I.‐Y. Jung , E. Noguera‐Ortega , R. Bartoszek , et al., “Tissue‐Resident Memory CAR T Cells With Stem‐Like Characteristics Display Enhanced efficacy Against Solid and Liquid Tumors,” Cell Reports Medicine 4, no. 6 (2023): 101053, 10.1016/j.xcrm.2023.101053. 37224816 PMC10313923

[50] N. N. Jarjour , T. S. Dalzell , N. J. Maurice , et al., “Collaboration Between Interleukin‐7 and ‐15 Enables Adaptation of Tissue‐Resident and Circulating Memory CD^8+^ T Cells to Cytokine Deficiency,” Immunity 58, no. 3 (2025): 616–631.e5, 10.1016/j.immuni.2025.02.009. 40023156 PMC13329436

[51] E. N. Arner and J. C. Rathmell , “Metabolic Programming and Immune Suppression in the Tumor Microenvironment,” Cancer Cell 41, no. 3 (2023): 421–433, 10.1016/j.ccell.2023.01.009. 36801000 PMC10023409

[52] M. J. Pittet , M. Di Pilato , C. Garris , and T. R. Mempel , “Dendritic Cells as Shepherds of T Cell Immunity in Cancer,” Immunity 56, no. 10 (2023): 2218–2230, 10.1016/j.immuni.2023.08.014. 37708889 PMC10591862

[53] X. Xiang , J. Wang , D. Lu , and X. Xu , “Targeting Tumor‐Associated Macrophages to Synergize Tumor Immunotherapy,” Signal transduction and targeted therapy 6, no. 1 (2021): 75, 10.1038/s41392-021-00484-9. 33619259 PMC7900181

[54] S. You , S. Li , L. Zeng , et al., “Lymphatic‐localized Treg‐mregDC Crosstalk Limits Antigen Trafficking and Restrains Anti‐tumor Immunity,” Cancer Cell 42, no. 8 (2024): 1415–1433.e12, 10.1016/j.ccell.2024.06.014. 39029466

