# Supplementary material for: CCL3 and IL‐7 Synergistically Enhance CAR‐T Efficacy in Solid Tumors
Source: Adv Sci (Weinh). 2026 Jun 22:e75993. Online ahead of print. doi: 10.1002/advs.75993 (PMC13336563; doi:10.1002/advs.75993)
Supplement: Supplementary file 1 — Supporting File 1: advs75993‐sup‐0001‐SuppMat.docx. [file ADVS-9999-e75993-s001.docx]

**Supplemental Information**

**CCL3 and IL-7 Synergistically Enhance CAR-T Efficacy in Solid Tumors**

Huanpeng Chen^1, 2, 9^, Xiaoyun Luo^1, 2, 9^, Huixin Gao^3, 9^, Yujing Ke^2^, Yu Zhan^4, 5^, Xiaoting Xu^4, 5^, Xiaoxiao Xiong^4, 5^, Fengjiao Wei^4, 5^, Si Yang^6^, Zhonghua Liu^7^, Bolan Yu^6^, Zhaofeng Huang^4,5,8 *^, Yingjie Bian^1, 2, *^

^
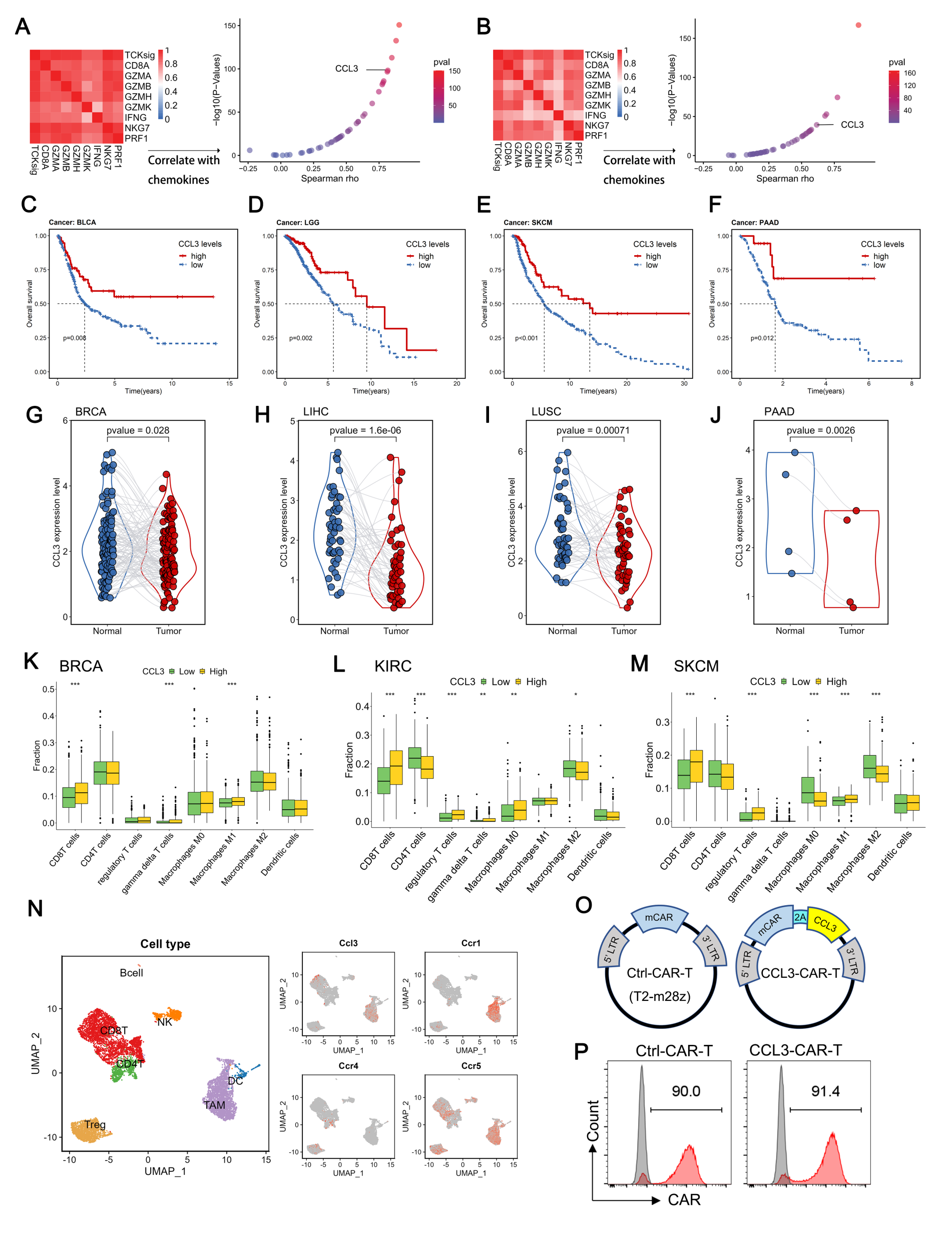
^

**Figure S1. CCL3 positively correlates with anti-tumor immunity in patients.**

**(A, B)** Spearman correlation of chemokines and TCKsig expression in Bladder urothelial carcinoma (BLCA, **A**, N=408) and Liver hepatocellular carcinoma (LIHC, **B**, N=371) from the TCGA database. **(C-F)** Association of CCL3 expression with patient survival in TCGA cohorts, including BLCA (**C**), Brain lower grade glioma (LGG, **D**), Skin cutaneous melanoma (SKCM, **E**) and Pancreatic adenocarcinoma (PAAD, **F**). **(G-J)** CCL3 expression levels in tumor and matched adjacent normal tissues from TCGA database, including Breast invasive carcinoma (BRCA, **G**), LIHC (**H**), Lung squamous cell carcinoma (LUSC, **I**) and (PAAD, **J**). **(K-M)** Deconvolution-based immune infiltration analysis of TCGA database reveals associations between CCL3 levels and immune cell proportions, including BRCA **(K)**, Kidney renal clear cell carcinoma (KIRC, **L)**, and SKCM **(M)**. **(N)** UMAP analysis of different immune cells and tumor cells from human skin cancer (MCC-GSE118056). Expression levels of CCL3 and its receptors CCR1, CCR4, and CCR5 in distinct cell populations. **(O)** The schemas diagram of murine CAR constructs is presented, including Ctrl-CAR (T2-m28z), and CCL3-expressing CAR (CCL3-CAR). **(P)** Flow cytometric analysis of the CAR-T cells transduced efficiency. The UTD were used as negative control. All data are presented as mean±SEM. Statistical significance: **p<0.05, **p<0.01, ***p<0.001, ****p<0.0001.* Log-rank (Mantel-Cox) test (C-F), Student’s t test (G-J), and two-way ANOVA with Tukey’s post-test (K-M).

**
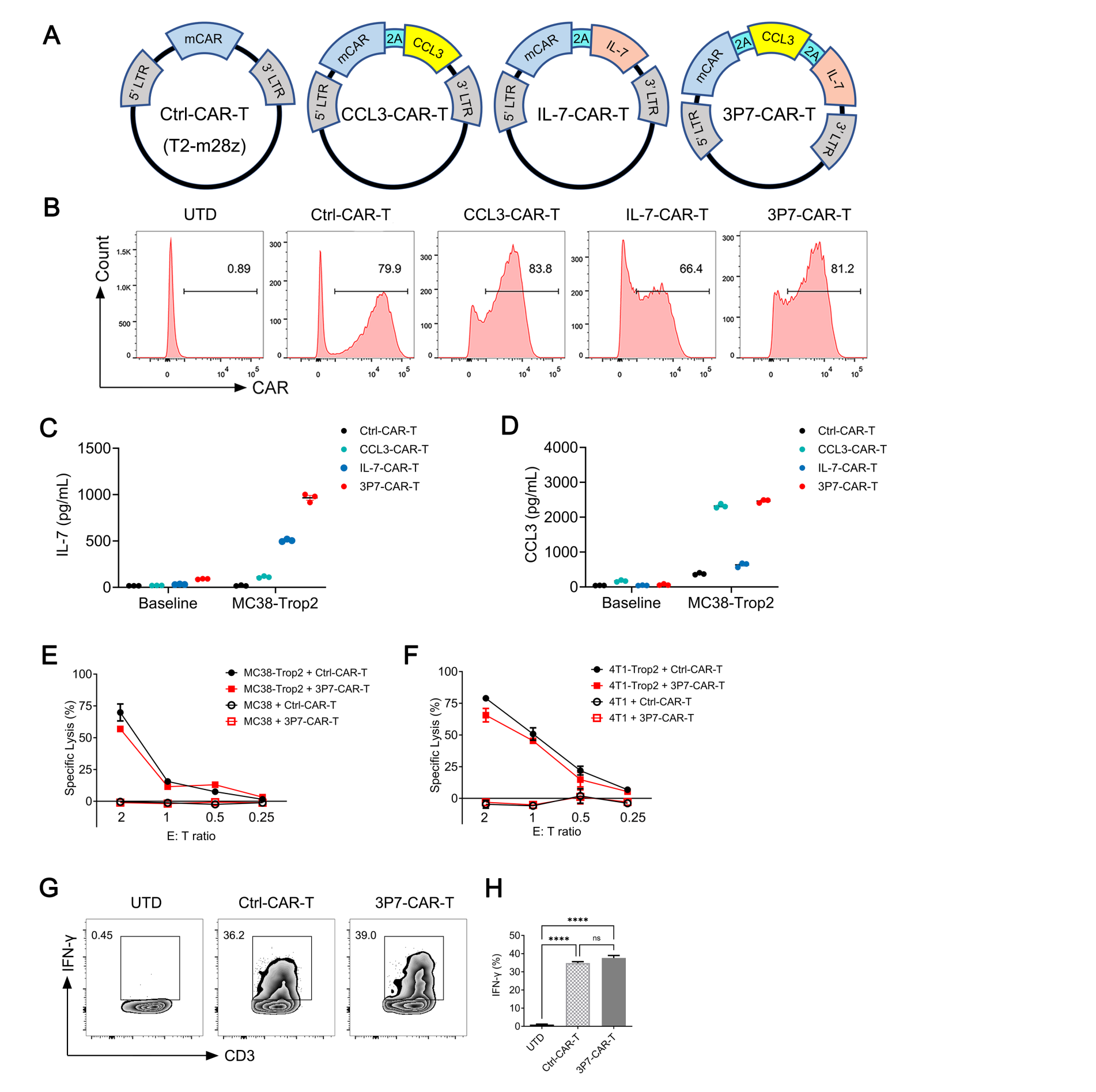
**

**Figure S2. 3P7-CAR-T cells maintain comparable cytolytic activity to control CAR-T cells in short-term tumor killing. (A)** The schemas diagram of second-generation murine CAR constructs, including control CAR (Ctrl-CAR-T), CCL3-expressing CAR (CCL3-CAR-T), IL-7-expressing CAR (IL-7-CAR-T), CCL3 and IL-7 co-expressing CAR (3P7-CAR-T). **(B)** Flow cytometric analysis of the CAR-T cells transduced efficiency. The UTD T cells were used as negative control. **(C, D)** The concentration of IL-7 **(C)** and CCL3 **(D)** in the supernatants of CAR-T cells cultured alone or after co-culture with MC38-Trop2 cells were determined by ELISA (N=3/group)**.** The LDH-release cytotoxicity against human Trop2 overexpression murine MC38-Trop2 cells **(E)** and 4T1-Trop2 cells **(F)** after 6h co-culture with CAR-T cells (N=3/group). **(G)** Flow cytometric analysis of IFN-γ^+^ CAR-T cells after 6h co-culture with MC38-Trop2 cells (N=3/group). Data are presented as mean ± SEM. Statistical significance: **p<0.05, **p<0.01, ***p<0.001, ****p<0.0001.* Two-way ANOVA with Tukey’s post-test (C-F), and One-way ANOVA with Tukey’s post-test (H).


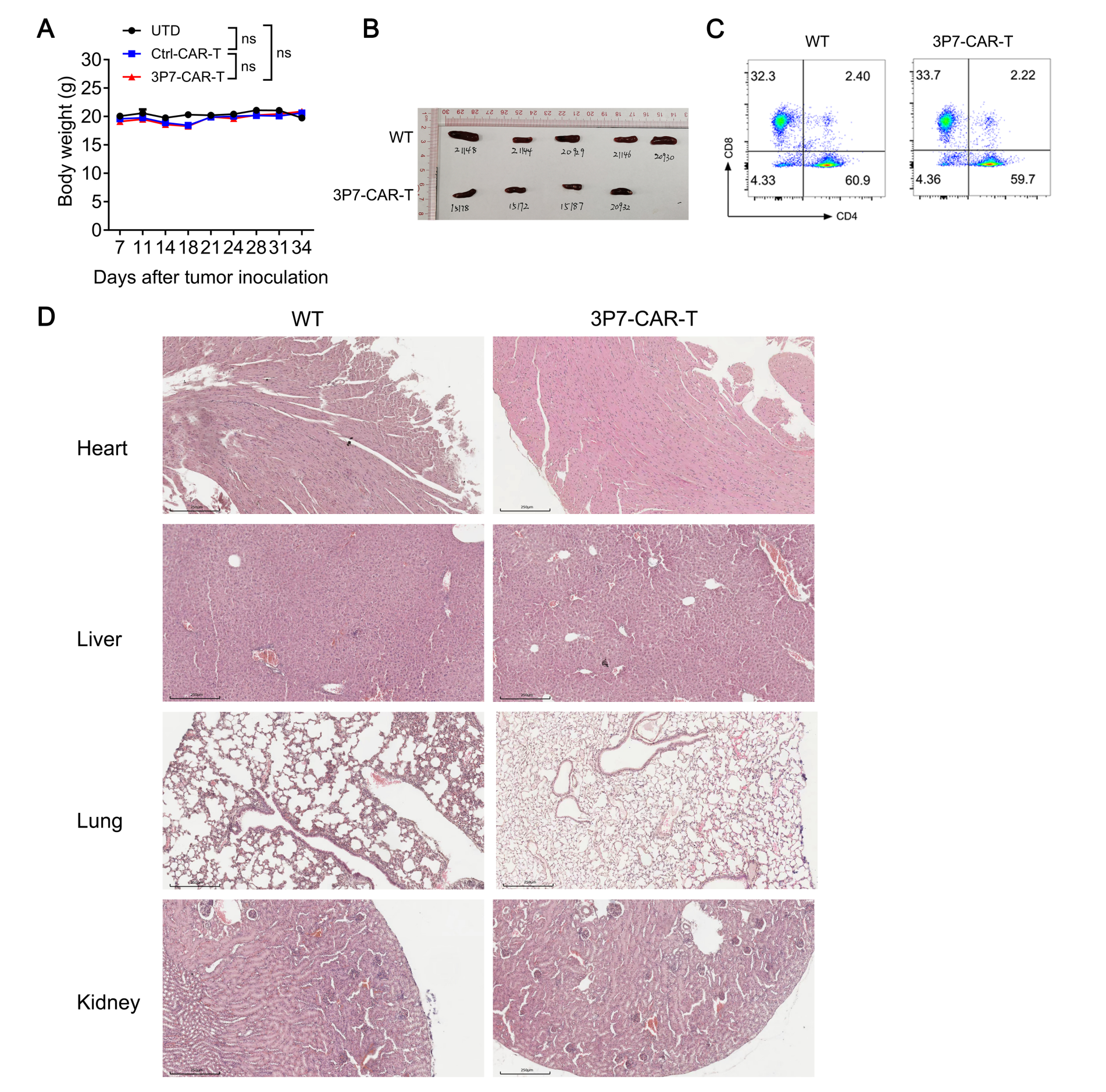


**Figure S3. 3P7-CAR-T therapy exhibits favorable safety without detectable toxic side effects.**

**(A)** The body weight of tumor-bearing mice in Figure 2I (N=5/group). **(B)** The photograph of resected spleen at experimental endpoint (day 18 post-tumor rechallenge) as indicated in Figure 5A. **(C)** Flow cytometric analysis of CD4^+^ and CD8^+^ T cell proportions in splenocytes from MC38-Trop2 bearing mice. **(D)** H&E staining of mouse major organs as indicated in Figure 2I. Scale bar, 250 μm. All data are presented as mean ± SEM. ns: no significance. One-way ANOVA with Tukey’s post-test (A).


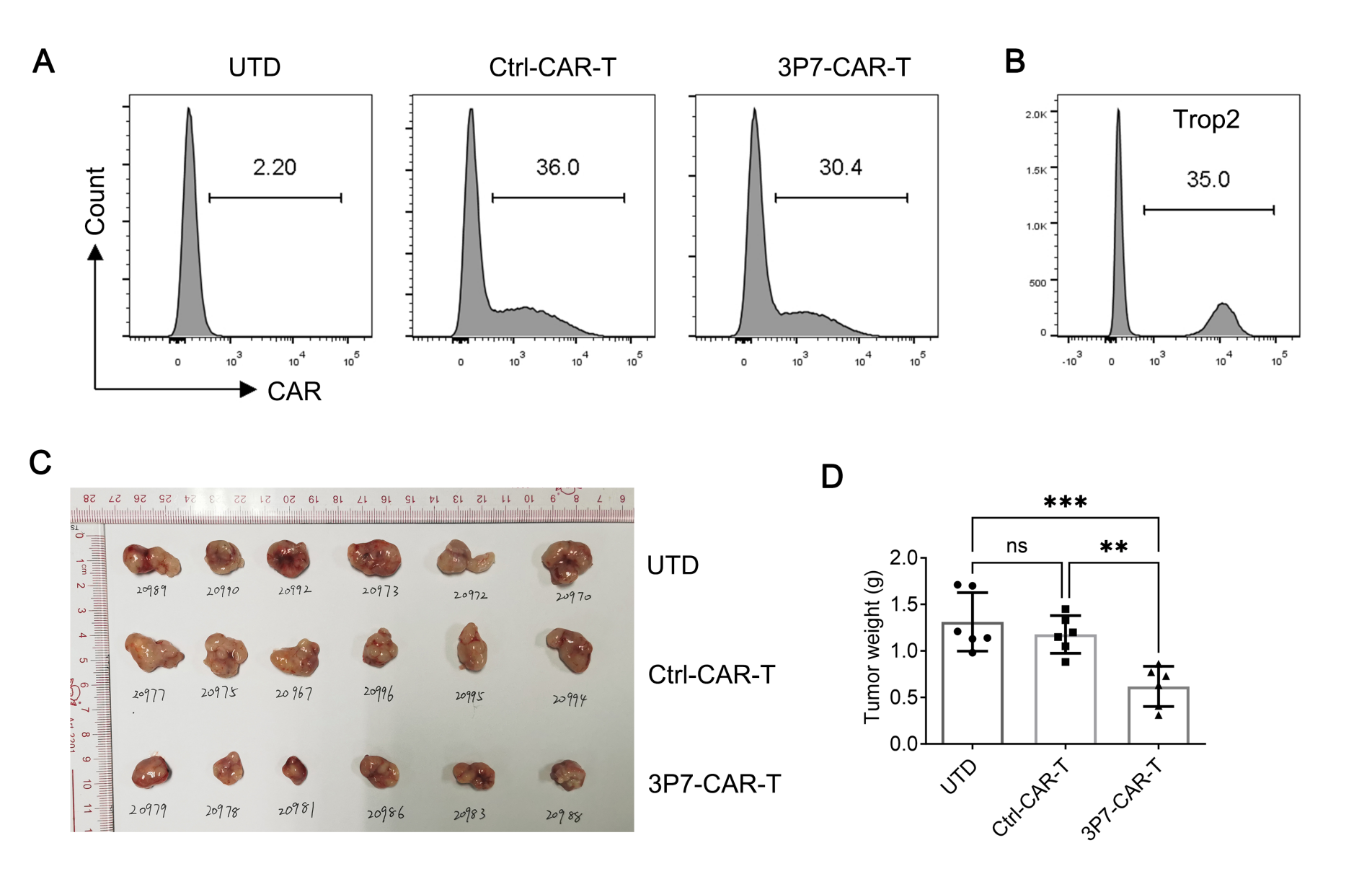


**Figure S4. 3P7-CAR-T cells improves anti-tumor effects in human colon cancer.**

**(A)** Flow cytometric analysis of the human Trop2-CAR-T cells transduced efficiency. The activated but untransduced T cells (UTD) were used as negative control. **(B)** Flow cytometric analysis of Trop2 expression on human colon cancer Colo-205 cells. **(C)** The image of resected human colon tumor mass at experimental endpoint as indicated in in Figure 3C. **(D)** The weights of individual tumors are presented (N=6/group). All data are presented as mean ± SEM. Statistical significance: **p<0.05, **p<0.01, ***p<0.001, ****p<0.0001.* One-way ANOVA with Tukey’s post-test (D).

**
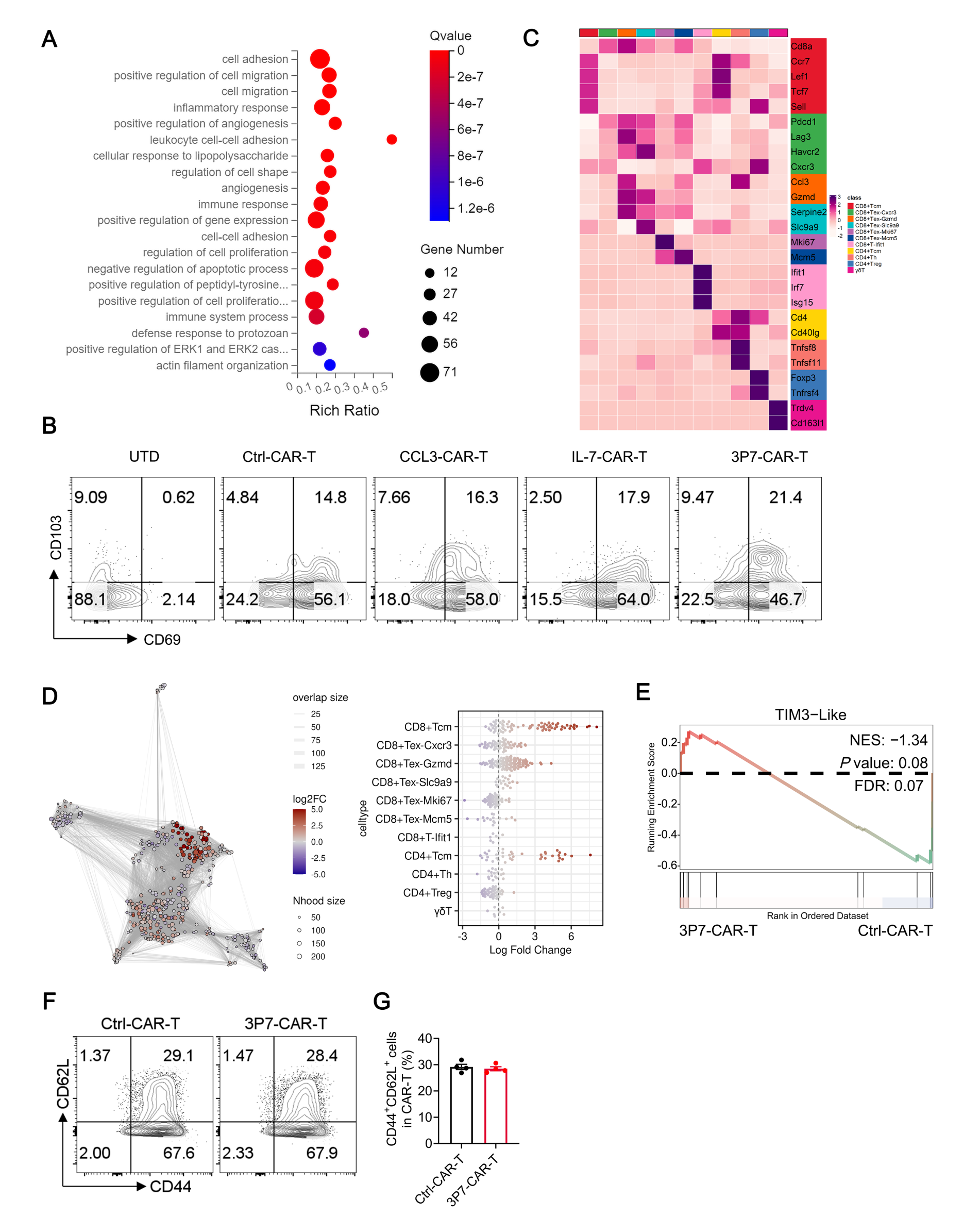
**

**Figure S5. 3P7-CAR-T cells induces memory T cells and endogenous anti-tumor response.**

**(A)** GO Biological Process enrichment analysis was performed for 7P3-CAR-T cells versus Ctrl-CAR-T control cells, and the bubble plot was generated. After co-culture CAR-T cells with MC38-Trop2 cells for 48 hours without cytokine supplementation, T cells were positively selected and subjected to RNA sequencing (n=3). **(B)** The representative images of CD69 and CD103 staining in CAR-T cells after co-culture with MC38-Trop2 cells by flow cytometry. **(C)** Heat-map showing the expression of signature genes of the 11 unique cluster of T cells as indicated in Figure 4G. **(D)** MiloR analysis of 11 unique populations identified among T immune cells in tumor. **(E)** GSEA analysis of Tim3-like gene sets in CD8 T cells from 3P7-CAR-T cells compared with Ctrl-CAR-T cells. **(F, G)** Representative images**(F)** and the proportion of CD44^+^CD62L^+^ cells in CAR-T cells **(G)** at the time of administration (N=4/group). Data are presented as mean ± SEM. Statistical significance: **p<0.05, **p<0.01, ***p<0.001, ****p<0.0001.* Student’s t test (G).


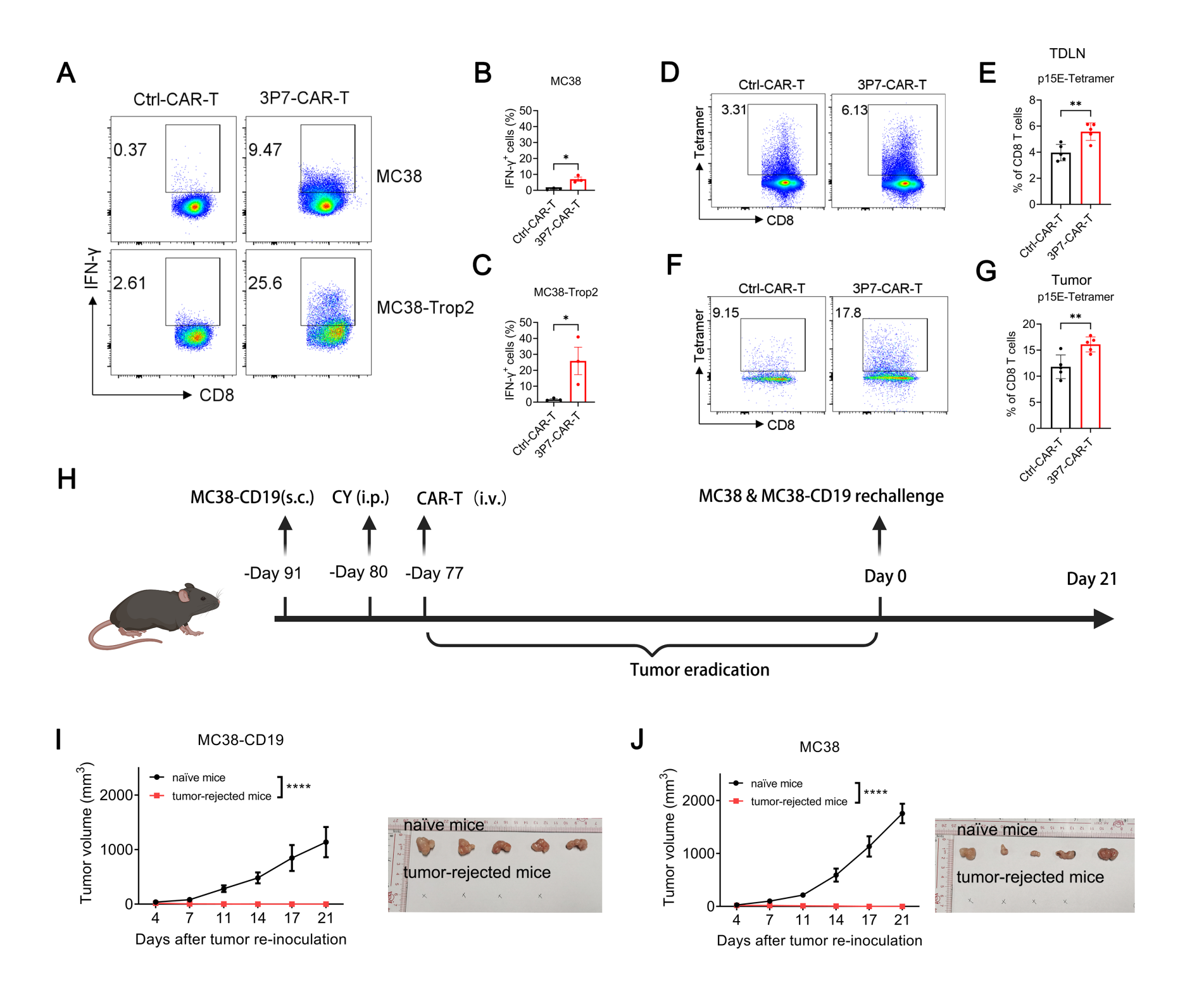


**Figure S6. 3P7-CAR-T cells induce both antigen and non-antigen specific memory responses.**

**(A-C)** Representative images and proportion of IFN-γ expression in TDLN CD8^+^ T cells **(A)** isolated from MC38-Trop2-bearing mice administered with Ctrl-CAR-T or 3P7-CAR-T, following 24 h of co-culture with MC38 **(B)** or MC38-Trop2 **(C)** tumor cells (N=3/group). **(D-G)** Representative image and proportion of p15E/H-2K^b^ tetramer positive cells in TDLN **(D, E)** and tumor-infiltrating lymphocytes **(F, G)** (N=5/group). **(H)** The schematic diagram of MC38/MC38-CD19 tumor re-challenge assay. Mice were inoculated with MC38-CD19. On day 11, mice were pretreated with CY, and 3 days later were administrated with CD19-3P7-CAR-T cells. At day 91 after CAR-T cell infusion, long-term surviving mice were re-challenged with a second inoculation of MC38-CD19 cells on the right flank, and parental MC38 cells on the left flank (N=4). As control, naïve C57 mice were inoculated with the same paradigm (N=5). **(I)** Tumor growth curve of MC38-CD19 on naïve mice and CD19-3P7-CAR-T tumor rejected mice. **(J)** tumor growth of MC38 on naïve mice, and CD19-3P7-CAR-T tumor rejected mice. Data are presented as mean ± SEM. Statistical significance: **p<0.05, **p<0.01, ***p<0.001, ****p<0.0001.* Student’s t test (B, C, E, and J), and two-way ANOVA with Tukey’s post-test (I, and J).

**
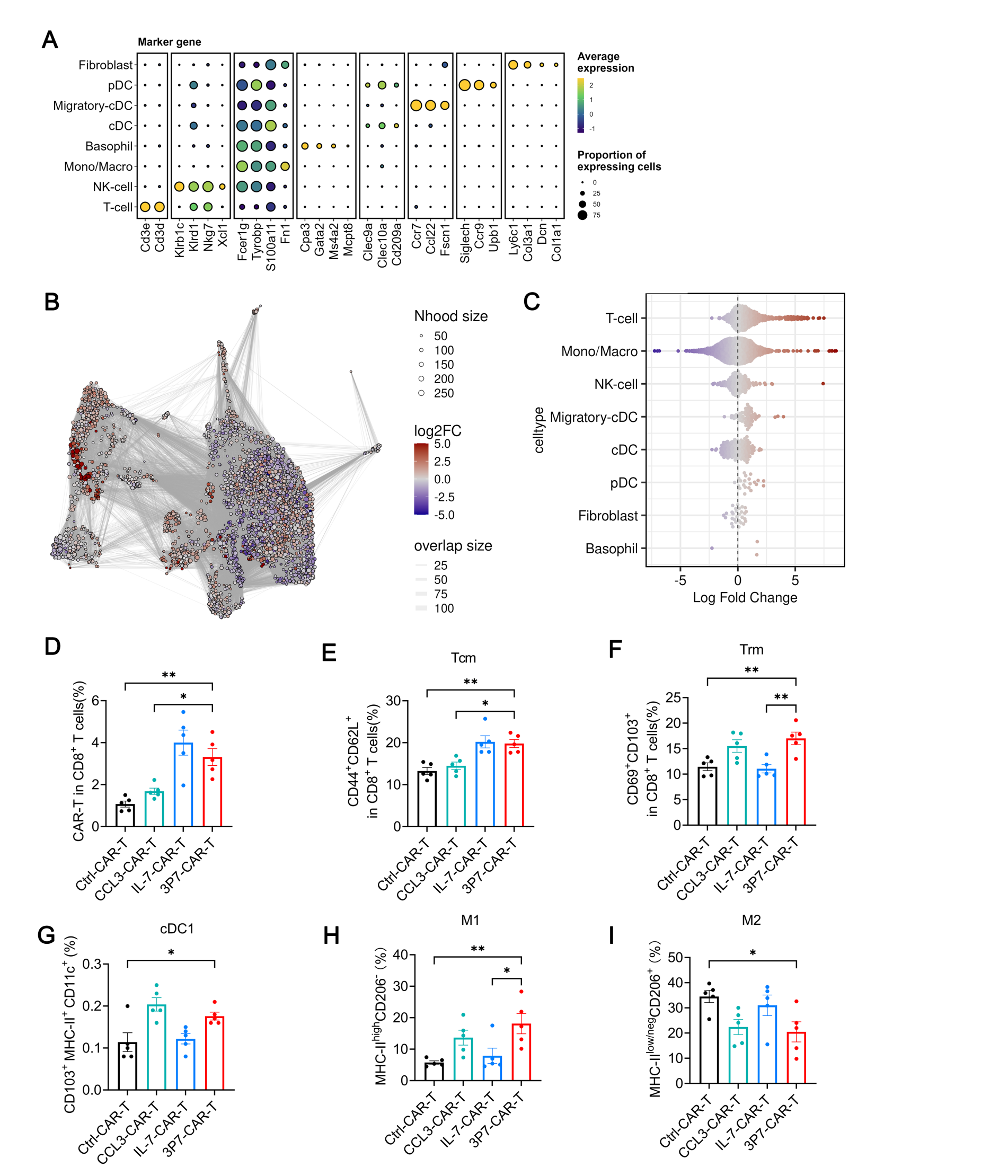
Figure S7. 3P7-CAR-T cells reprograms tumor immune microenvironment and activate endogenous anti-tumor responses.**

**(A)** Signature genes of the immune cell clusters from single cell RNA-seq data, cells were clustered with CD45^+^CD3^-^ cells. **(B)** MiloR analysis of the 8 unique clusters identified among CD45^+^CD3^-^ immune cells. **(C)** Clusters difference among 3P7-CAR-T compared with Ctrl-CAR-T groups. **(D-F)** Flow cytometric analysis of CD8^+^ T cells from MC38-Trop2 tumor-bearing mice treated with CAR-T cells, showing the proportions of CAR-T cells **(D)**, CD44^+^ CD62L^+^ cells **(E)** and CD69^+^CD103^+^ cells **(F)** in TILs**. (G)** Flow cytometric analysis of CD103^+^MHCII^+^CD11c^+^ DC1 among CD45^+^ cells in tumors from CAR-T-treated mice. **(H, I)** The proportion of M1-like (MHCII^high^CD206^-^) and M2-like (CD206^+^MHC-II^low/neg^) cells among CD45^+^CD3^-^CD11b^+^F4/80^+^ TAMs in CAR-T-treated mice (N=5/group). Data are presented as mean ± SEM. Statistical significance: **p<0.05, **p<0.01, ***p<0.001, ****p<0.0001.* One-way ANOVA with Tukey’s post-test (D-I).

**
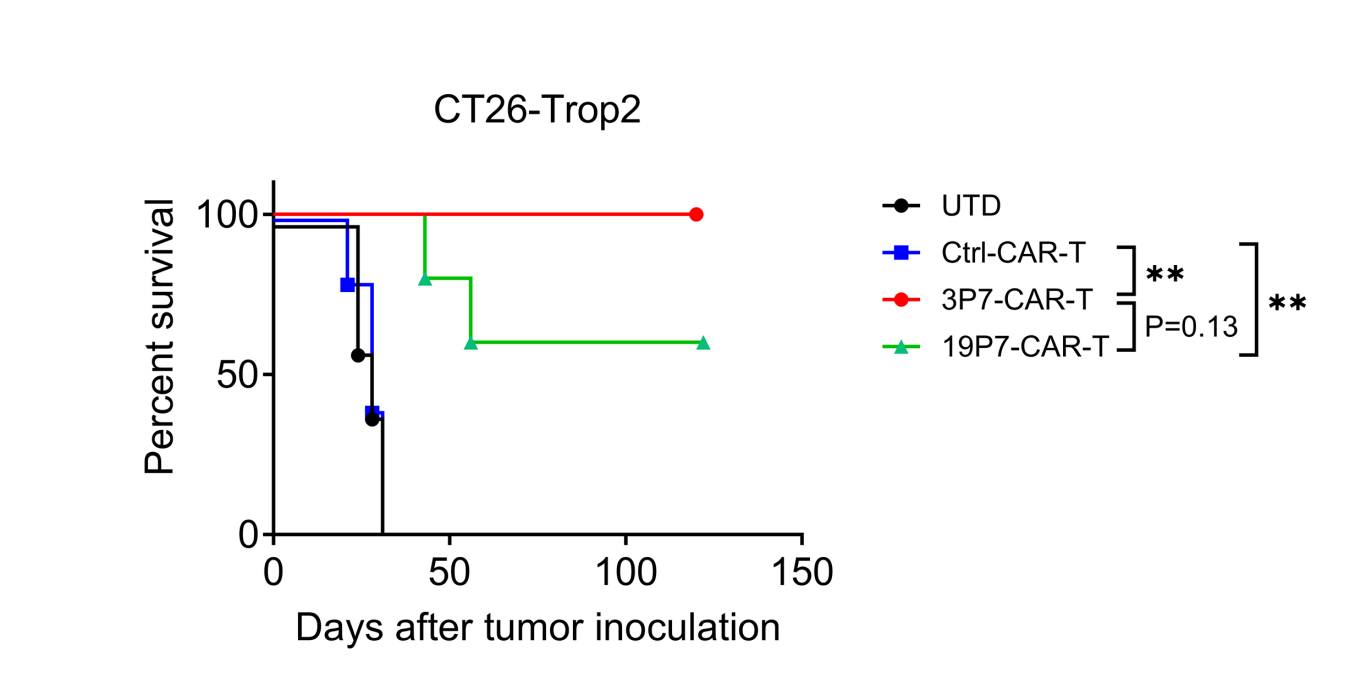
**

**Figure S8. Targeting CCL3 is preferable to CCL19 for cold tumor CAR-T therapy.**

Survival curves of CT26-Trop2 tumor-bearing mice pre-treated with CY (i.p., day 8 post-inoculation) followed by administration of Ctrl-CAR-T, 3P7-CAR-T, or 19P7-CAR-T cells on day 11. N=5 group. Survival rates were compared using Log-rank (Mantel-Cox) test. Data are presented as mean ± SEM. Statistical significance: **p<0.05, **p<0.01, ***p<0.001, ****p<0.0001.* Log-rank (Mantel-Cox) test.

**Table S1: Top 10 TCRβ clonotypes after 3P7-CAR-T treatment.**

| **TCR amino acid sequence** | **V gene** | **J gene** | **Clone count** | **Frequency (%)** | **Rank** | **p15E TCRβ identity (%)** |
| --- | --- | --- | --- | --- | --- | --- |
| TCRα: CALNNYAQGLTF;  TCRβ: CASSLLDSSNERLFF | TRAV13-1; TRBV29 | TRAJ26; TRBJ1-4 | 63 | 6.65 | 1 | 68.75 |
| TCRα: CAMRGGTEGADRLTF;  TCRβ: CASRERDWSSYEQYF | TRAV16; TRBV15 | TRAJ45; TRBJ2-7 | 50 | 5.27 | 2 | 37.50 |
| TCRα: CALGVNNYAQGLTF;  TCRβ: CASSFLTGGNSDYTF | TRAV6N-7; TRBV29 | TRAJ26; TRBJ1-2 | 31 | 3.27 | 3 | 37.50 |
| TCRα: CALSNYNVLYF;  TCRβ: CASSFLQGADTEVFF | TRAV13-1; TRBV29 | TRAJ21; TRBJ1-1 | 30 | 3.16 | 4 | 43.75 |
| TCRα: CATDNAGAKLTF;  TCRβ: CASSFGGTNERLFF | TRAV8-1; TRBV29 | TRAJ39; TRBJ1-4 | 22 | 2.32 | 5 | 75.00 |
| TCRα: CAASRTQVVGQLTF;  TCRβ: CASSFGGEGERLFF | TRAV7D-2; TRBV29 | TRAJ5; TRBJ1-4 | 13 | 1.37 | 6 | 68.75 |
| TCRα: CALGANNYAQGLTF;  TCRβ: CASSFLDGNYAEQFF | TRAV6N-7; TRBV29 | TRAJ26; TRBJ2-1 | 13 | 1.37 | 7 | 43.75 |
| TCRα: CAFNNNAGAKLTF;  TCRβ: CASSDNANSDYTF | TRAV13-1; TRBV13-3 | TRAJ39; TRBJ1-2 | 10 | 1.05 | 8 | 25.00 |
| TCRα: CAVSAPTEGADRLTF;  TCRβ: CASSLQGDTQYF | TRAV3-3; TRBV16 | TRAJ45; TRBJ2-5 | 10 | 1.05 | 9 | 31.25 |
| TCRα: CVLARNNYAQGLTF;  TCRβ: CASSFLDNNQDTQYF | TRAV6-1; TRBV29 | TRAJ26; TRBJ2-5 | 10 | 1.05 | 10 | 31.25 |

Note: p15E TCRβ sequence is CASSQGGGISNERLFF.
