# Supplementary figures and images for: CCL3 and IL‐7 Synergistically Enhance CAR‐T Efficacy in Solid Tumors
Source: Adv Sci (Weinh). 2026 Jun 22:e75993. Online ahead of print. doi: 10.1002/advs.75993 (PMC13336563; doi:10.1002/advs.75993)

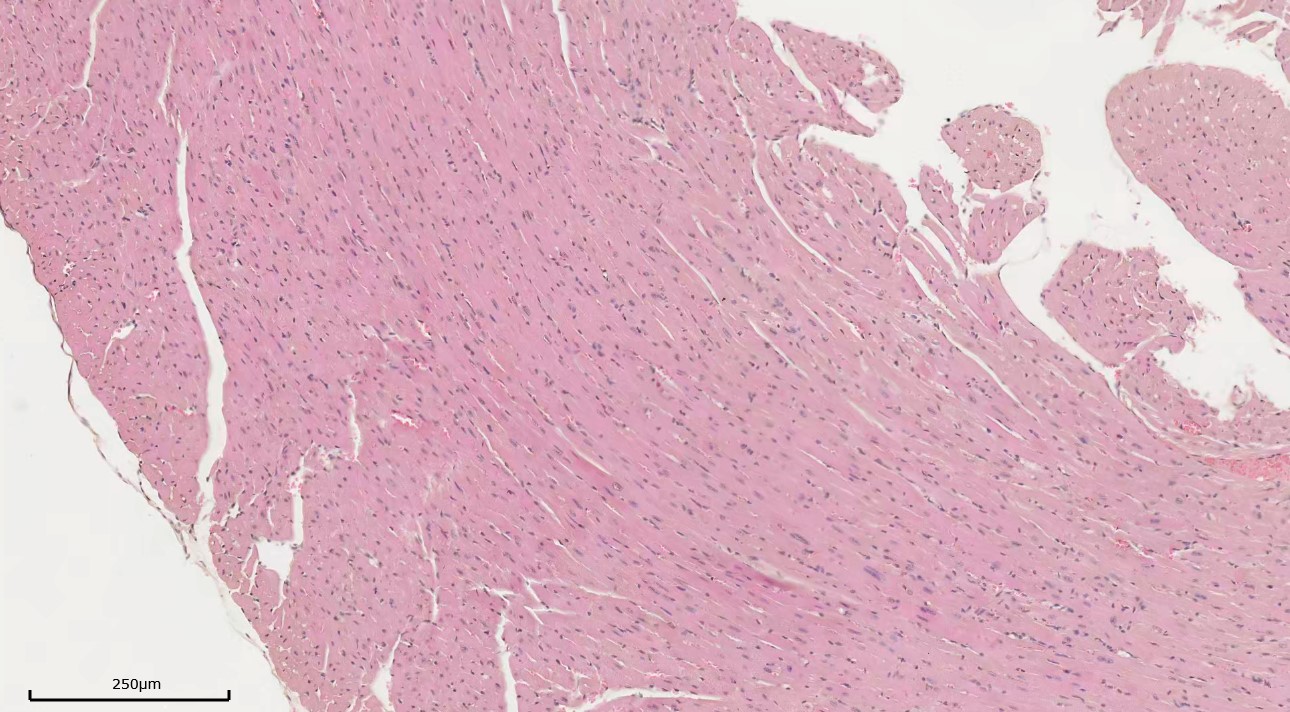

Supplement: Supplementary file 2 — Supporting File 2: advs75993‐sup‐0002‐Data.zip. [file ADVS-9999-e75993-s002.zip › advs75993-sup-0002-Data/FigureS3-HE/3P7-heart_10.00X.png]

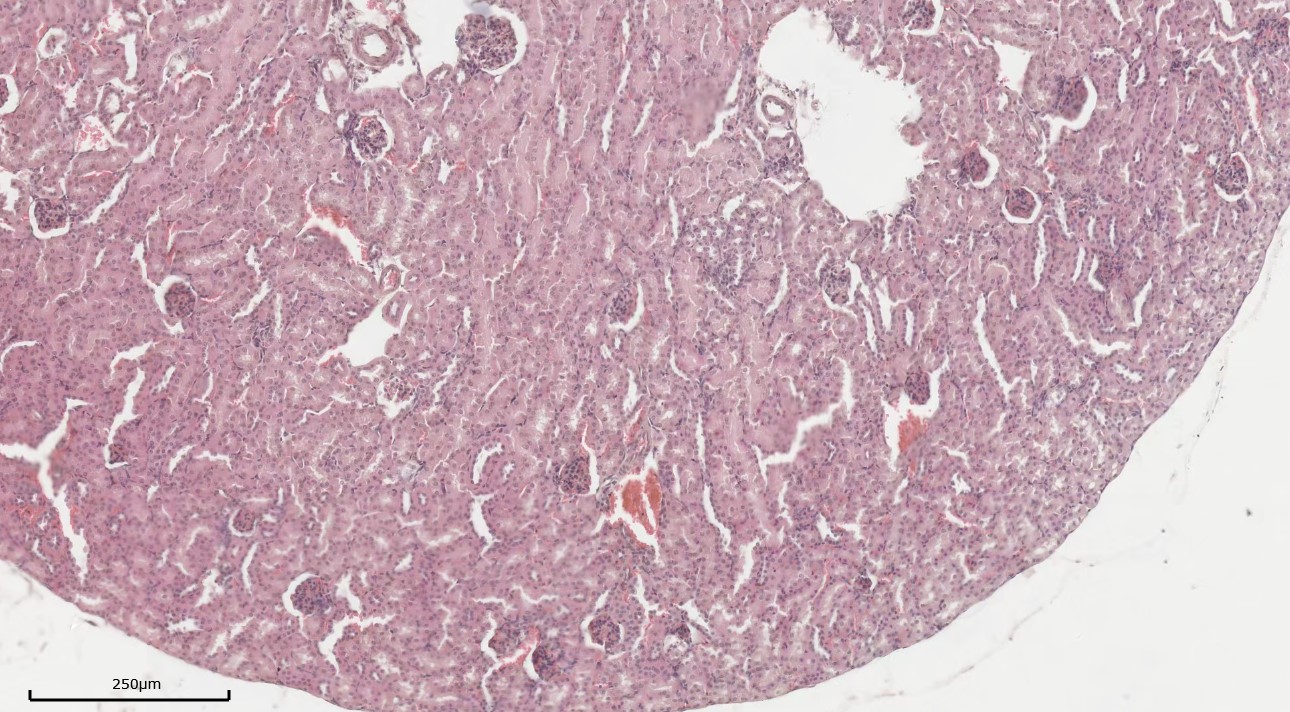

Supplement: Supplementary file 2 — Supporting File 2: advs75993‐sup‐0002‐Data.zip. [file ADVS-9999-e75993-s002.zip › advs75993-sup-0002-Data/FigureS3-HE/3P7-kidney_10.00X.png]

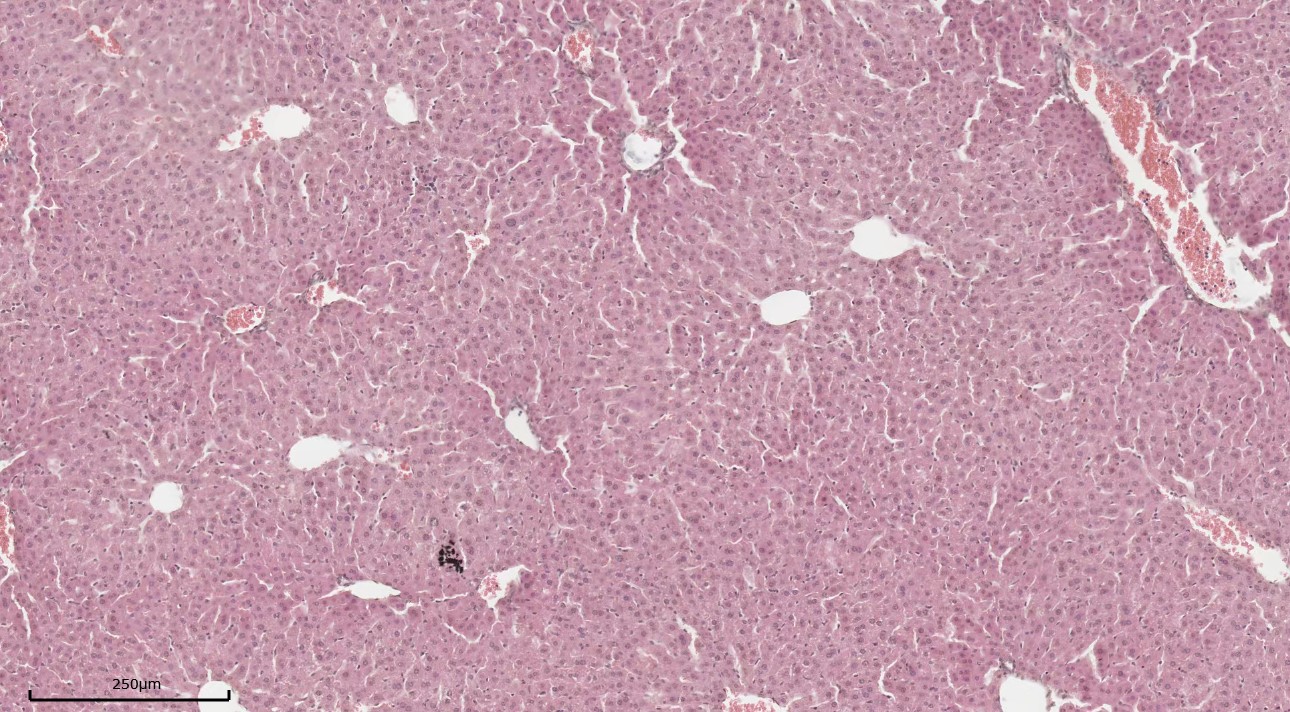

Supplement: Supplementary file 2 — Supporting File 2: advs75993‐sup‐0002‐Data.zip. [file ADVS-9999-e75993-s002.zip › advs75993-sup-0002-Data/FigureS3-HE/3P7-liver_10.00X.png]

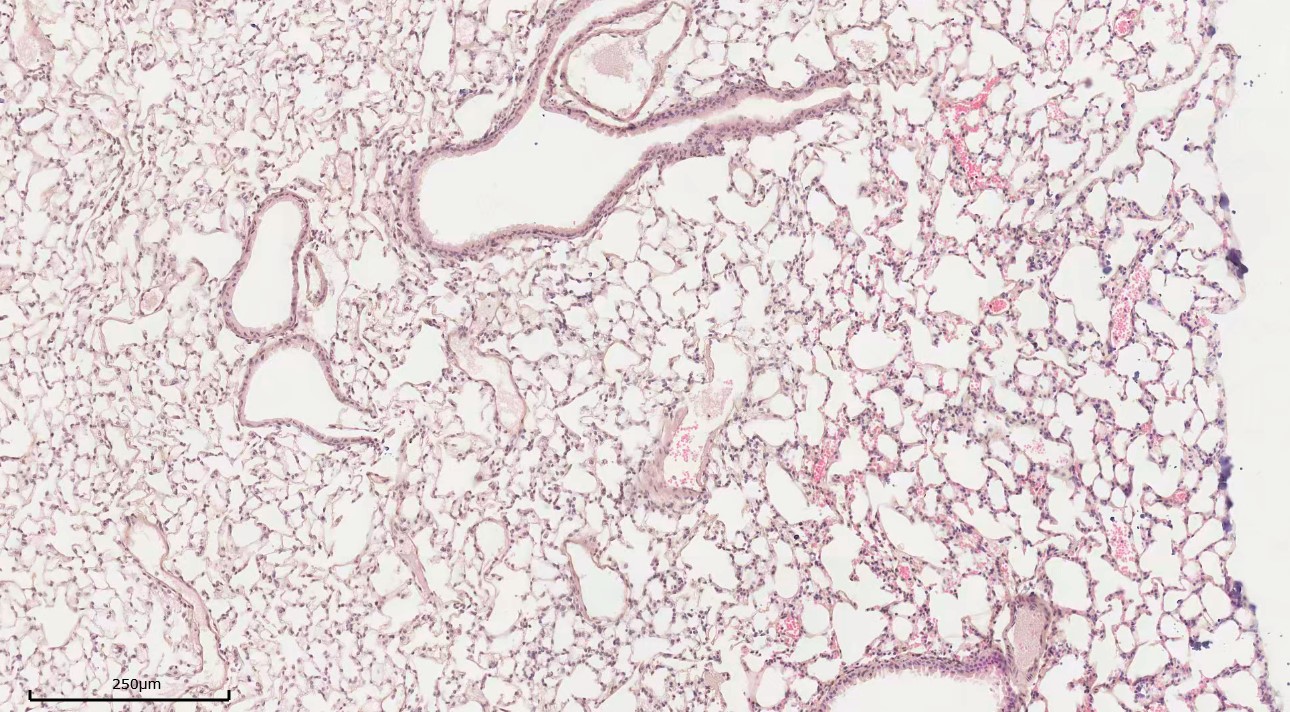

Supplement: Supplementary file 2 — Supporting File 2: advs75993‐sup‐0002‐Data.zip. [file ADVS-9999-e75993-s002.zip › advs75993-sup-0002-Data/FigureS3-HE/3P7-lung_10.00X.png]

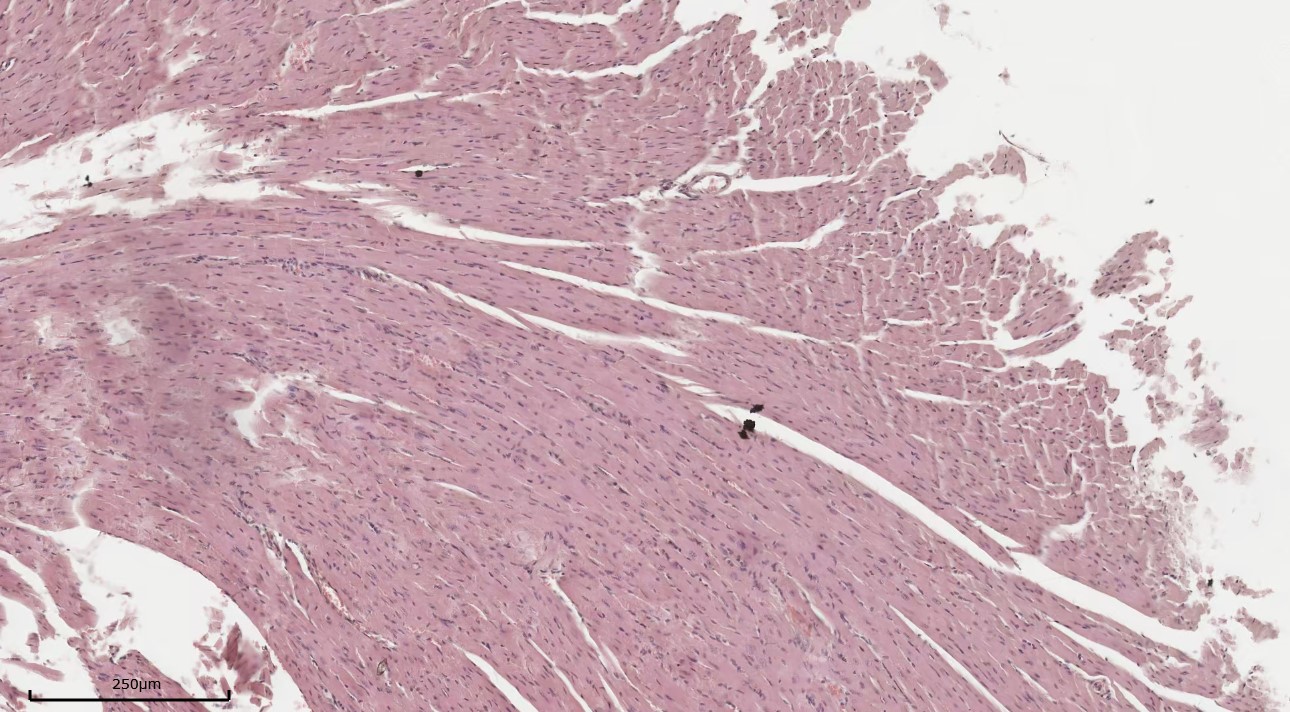

Supplement: Supplementary file 2 — Supporting File 2: advs75993‐sup‐0002‐Data.zip. [file ADVS-9999-e75993-s002.zip › advs75993-sup-0002-Data/FigureS3-HE/WT-heart_10.00X.png]

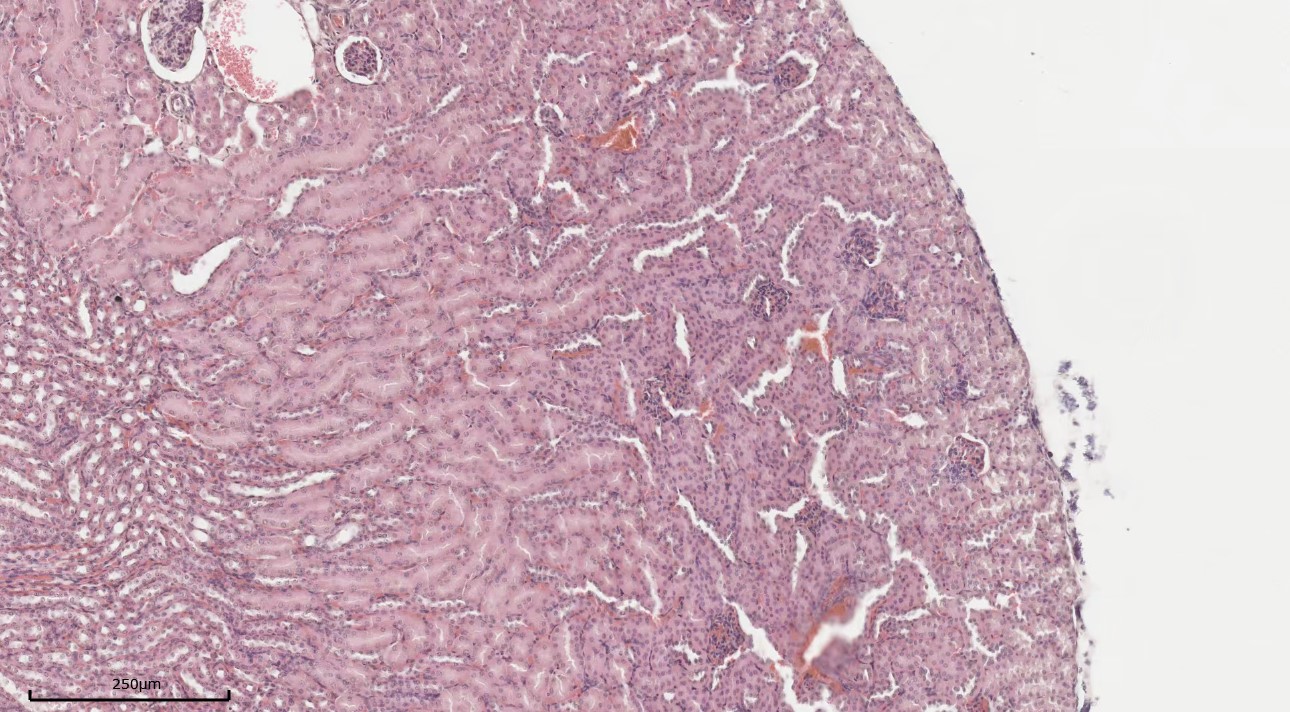

Supplement: Supplementary file 2 — Supporting File 2: advs75993‐sup‐0002‐Data.zip. [file ADVS-9999-e75993-s002.zip › advs75993-sup-0002-Data/FigureS3-HE/WT-kidney_10.00X.png]

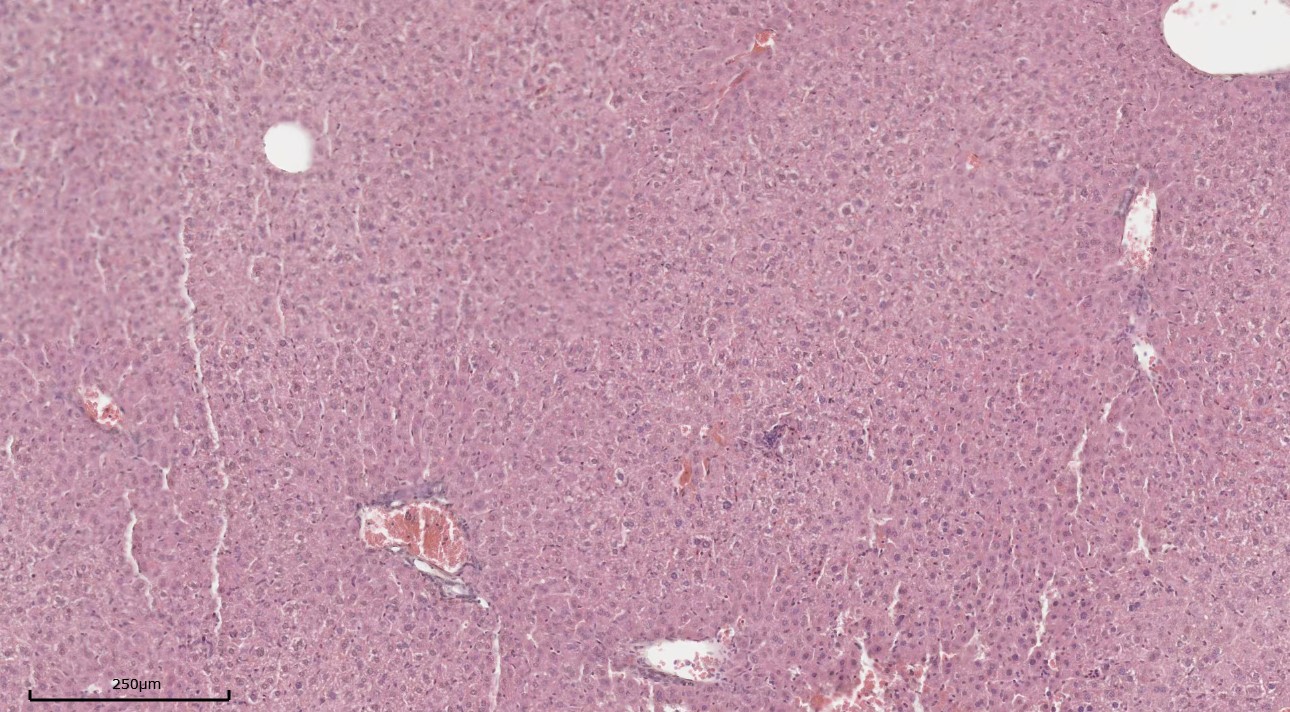

Supplement: Supplementary file 2 — Supporting File 2: advs75993‐sup‐0002‐Data.zip. [file ADVS-9999-e75993-s002.zip › advs75993-sup-0002-Data/FigureS3-HE/WT-liver_10.00X.png]

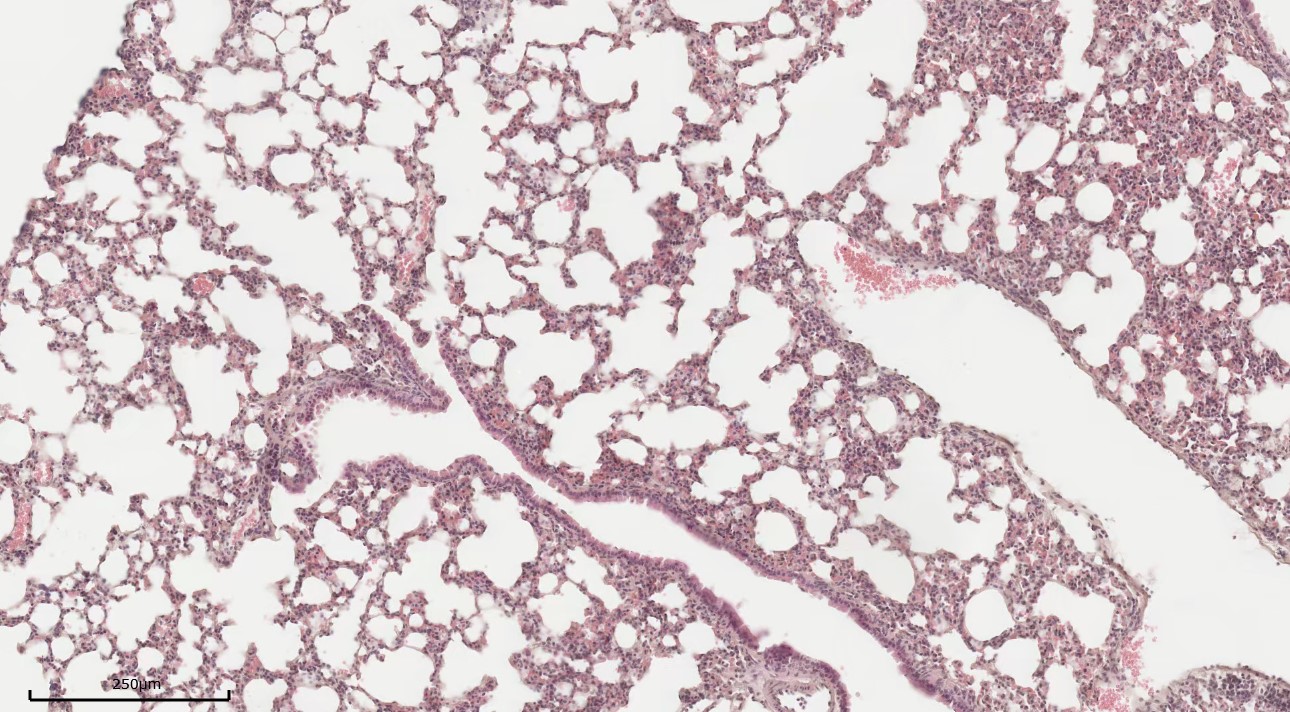

Supplement: Supplementary file 2 — Supporting File 2: advs75993‐sup‐0002‐Data.zip. [file ADVS-9999-e75993-s002.zip › advs75993-sup-0002-Data/FigureS3-HE/WT-lung_10.00X.png]
